# Abstracts of the 11^th^ International Conference on Cachexia, Sarcopenia and Muscle Wasting, Maastricht, The Netherlands, 7–9 December 2018 (Part 2)

**DOI:** 10.1002/jcsm.12407

**Published:** 2019-03-28

**1-07**

**Skeletal muscle bioenergetics in critically ill: effect of early rehabilitation on mitochondrial function and insulin resistance during and 6 months after critical illness**

**Tomáš Urban**, Adéla Krajčová, Petr Waldauf, Barbora Blahutová, Jan Gojda and František Duška

*OXYLAB – Department of Anaesthesia and Intensive Care Medicine, 3rd Faculty of Medicine, Kralovské Vinohrady University Hospital, Prague, Czech Republic*

**Introduction:** The hallmark of metabolic changes in skeletal muscle during critical illness is impaired aerobic phosphorylation in mitochondria^1^ and reduced insulin‐stimulated glucose disposal.^2^ We asked whether these parameters can be influenced by very early (started <48 h) rehabilitation using functional‐electrical stimulation assisted supine cycling (FESCE).

**Methods:** In a nested subgroup of patients in a prospective randomized clinical trial of early rehabilitation (NCT 02864745), we performed serial *vastus lateralis* muscle biopsies and euglycemic hyperinsulinaemic (120 mIU.m^−2^ BSA.min^−1^) clamps at Days 0, 7, and 180. Mitochondrial function was assessed by high resolution respirometry from native skeletal muscle homogenates as described,^3^ with a cohort (*n* = 8) of metabolically healthy age‐matched elective hip surgery patient serving as the control group. Electron flux through mitochondrial respiratory complexes was measured by addition of specific substrates and inhibitors described in.^3^

**Results and Discussion:** In the control group, the mean rehabilitation dose was 22 min a day, whilst interventional group was receiving 77 min/day (*P* < 0.01). *Insulin resistance*: Glucose disposal was lowest in the acute phase of critical illness (1.53 ± 0.99 vs. 1.21 ± 0.92 mmol/min) and improved a little after 7 days in both groups (to 2.23 ± 1.01 vs. 2.05 ± 0.82 mmol/min) and after 6 months (3.32 ± 0.59 vs. 2.72 ± 0.90 mmol/min). ***Bioenergetic function***: In keeping with previous studies, critical illness led to a mild impairment of aerobic phosphorylation, with major defect being in respiratory Complex I and II, whilst fatty acid oxidation was upregulated. In standard rehabilitation group, this pattern persisted up until 6 months after the critical illness, whilst in the early rehabilitation group, it seems to normalize or even overshoot to supranormal values. The major limitation indeed is the low number of subjects accumulated so far in this ongoing study. This is the reason why these data are to be considered preliminary and have not been formally statistically processed.

**In conclusion,** our preliminary data show that critical illness leads to profound changes in skeletal muscle bioenergetics, which seem to persist in survivors at least 6 months but could be influenced by early rehabilitation.

**Table:** 

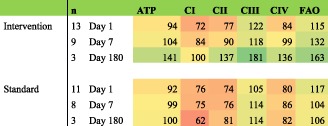

Table: Mitochondrial functional indices expressed as % of values in the control group. Note: ATP, aerobic phosphorylation.

References1

Jiroutkova, K
 et al.: Crit Care
2015;19:448
10.1186/s13054-015-1160-x
26699134PMC46993392

Bakalar
B
 et al: Crit Care Med
2006; 34(2):381–386: https://www.ncbi.nlm.nih.gov/pubmed/16424718
1642471810.1097/01.ccm.0000196829.30741.d43

Ziak
J
 et al: Mitochondrion
2015; Mar 21:106–112
https://www.ncbi.nlm.nih.gov/pubmed/25701243
10.1016/j.mito.2015.02.00225701243

**1-09**

**Decubitus abdominal circumference cut‐off points for low BMI**

**Alejandro Sanz‐Paris** ^1,2^, Javier Perez‐Nogueras^3^, Maria Martinez‐García^1^, Maria‐Elena Lopez‐Alaminos^1^, M. Gonzalez‐Fernandez^1^, A. Sanz‐Arque^4^ and Jose M. Arbones‐Mainar^2,5,6^

^1^ *Nutrition Unit, Miguel Servet Hospital, Zaragoza, Spain;* ^2^ *Research institute in Zaragoza, Instituto de Investigación Sanitaria Aragón (IIS‐Aragón), Zaragoza, Spain;* ^3^ *Geriatric Unit, Tudela, Spain;* ^4^ *General Practitioner, Tudela Health Center, Tudela, Spain;* ^5^ *Adipocyte and Fat Biology Laboratory (AdipoFat), Translational Research Unit, University Hospital Miguel Servet, Instituto Aragonés de Ciencias de la Salud (IACS), Zaragoza, Spain;* ^6^ *Centro de Investigación Biomédica en Red Fisiopatología Obesidad y Nutricion (CIBERObn), Instituto Salud Carlos III, Madrid, Spain*

The most extended anthropometric parameter for undernutrition is BMI < 18.5 kg/m^2^ followed by calf and the mid‐upper arm circumference. This study was conducted with the main purpose to assess which anthropometric parameter better represented this BMI value. Furthermore, we aimed to obtaining the cut‐off points for a BMI < 18.5 kg/m^2^.

**Material and methods:** Transversal observational study carried out in patients assisted in the matter of undernutrition (*n* = 158). Anthropometric parameters and the BMI were obtained. Skeletal muscular mass was estimated using the Jenssen formula via electrical impedance; and the MMI, low MMI, and fat mass percentage variables were gathered. SPSS 24 was used. The differences of the variables depending on gender were analysed using the *T* of Student or the *U* of Mann–Whitney. The association between variables was analysed through the Pearson or Spearman correlation, and the comparison between qualitative variables was undertaken by using the chi‐square test. Statistical significance was reached with *P* < 0.05. Local Ethical Committee approved.

**Results:** In women, the BMI is more highly correlated with waist circumference in decubitus and fat mas percentage (*r* = 0.58 and *r* = 0.578 years, respectively). In men, BMI is correlated again with waist circumference in decubitus(*r* = 0.72) and brachial circumference (*r* = 0.6) and fat mass percentage (*r* = 0.26). By applying COR curves, the waist circumference was a better predictor of a BMI inferior to 18.5 kg/m^2^ (cut‐off points of 77.9 cm in women and 76.5 cm in men) than the other anthropometric parameters.

**Conclusions:** The waist circumference is classically associated to high BMI in obesity. Its determination in decubitus could be a better predictor of low BMI than the calf and brachial circumferences. Cut‐off waist circumference for high BMI is well recognized but not for low BMI.

**1-31**

**Characterization of non‐responders to a resistance exercise program in old adults**

**Alfons Ramel** ^1,2^, Olof G. Geirsdottir^1,2^, Milan Chang^1,3^, Kristin Briem^4^, Palmi V. Jonsson^1,5^ and Inga Thorsdottir^6^

^1^ *The Icelandic Gerontological Research Center, Reykjavik, Iceland;* ^2^ *Faculty of Food Science and Nutrition, University of Iceland, Reykjavik, Iceland;* ^3^ *Sport Science, School of Science and Engineering, Reykjavik University, Reykjavik, Iceland;* ^4^ *Department of Physiotherapy, University of Iceland, Reykjavik, Iceland;* ^5^ *Department of Geriatrics, National University Hospital of Iceland, Reykjavik, Iceland;* ^6^ *School of Health Sciences, University of Iceland, Reykjavik, Iceland*

**Background:** Sarcopenia contributes to functional impairment, frailty, and disability. Resistance exercise is recommended to prevent or delay sarcopenia. The aim of this study was to characterize old adults who did not respond to a resistance exercise program.

**Methods:** Community dwelling participants (*N* = 236, 73.7 ± 5.7 years, 58.2% female) participated in a supervised 12‐week resistance exercise program (REP). Body composition (DXA), quadriceps strength, 6‐min‐walk for‐distance (6MWD), timed‐up‐and‐go performance (TUG), dietary intake, and background variables were assessed. Non‐responders were defined as not having gained appendicular muscle mass.

**Results:** Two hundred and eleven (90.3%) participants completed the REP. Responders (80.1%) increased lean mass (1.1 ± 1.1 kg), whereas non‐responders (19.9%) decreased lean mass (−0.3 ± 1.4 kg, *P* = 0.001). No significant baseline differences between the two groups were found in age, BMI, body composition, quadriceps strength, 6MWD, TUG, or medication. However, participants who lost lean mass had a significant lower energy intake (1551 ± 394 vs. 1724 ± 490 kcal/day, *P* = 0.046) and lower protein intake (0.86 ± 0.24 vs. 0.98 ± 0.27 g protein/kg body, *P* = 0.009). According to regression analysis corrected for potential confounders, an additional protein intake of 0.1 g protein/body weight increased the likelihood for a positive training response by 28% (*P* = 0.006).

**Conclusions:** Our study shows that energy and dietary protein intake were positively associated with an increase of appendicular muscle mass in older adults participating in REP. The results further indicate that a protein intake higher than 0.8 g/kg per day is necessary to achieve optimal response to training.

**1-32**

**Obesity, physical function, and wrist fracture risk in community dwelling adults**

**Alfons Ramel** ^1,2^, Bergthora Baldursdottir^2,3,4^, Hannes Petersen^2,5^, Palmi V. Jonsson^2,3^, Brynjolfur Mogensen^2^, Susan L. Whitney^6^ and Ella K. Kristinsdottir^2^

^1^ *Facultion of Food Science and Nutrition, University of Iceland, Reykjavik, Iceland;* ^2^ *The Icelandic Gerontological Research Centre, Reykjavik, Iceland;* ^3^ *Faculty of Medicine, University of Iceland, Reykjavik, Iceland;* ^4^ *Department of Physiotherapy, Landspitali, University Hospital of Iceland, Reykjavik, Iceland;* ^5^ *Akureyri Hospital, Akureyri, Iceland;* ^6^ *Department of Physical Therapy, University of Pittsburgh, Pittsburgh, PA, USA*

**Background:** Wrist fracture is the most common first fracture and is known to be a strong predictor of hip fracture. The aim of this study was to investigate whether higher BMI is related to wrist fractures in middle aged and old Icelandic adults.

**Methods:** A case control study was conducted with 98 subjects aged 50–75 years having sustained a fall‐related wrist fracture. Forty eight sex‐, age‐, and physical activity‐matched individuals without previous wrist fracture served as controls. Measurements included: Head‐Shake Test, tuning fork, biothesiometer, Semmes‐Weinstein monofilaments, Sensory Organization Test, Five‐times‐sit‐to‐stand test (FTSTS), 10‐m‐walk test, the Activities‐Specific Balance Confidence, and the Dizziness Handicap Inventory scales.

**Results:** Normal weight participants (*n* = 50) showed better physical function (*P* < 0.05) than their overweight (*n* = 66) and obese counterparts (*n* = 32). However, there were no differences in sensory function. Balance was higher and dizziness was lower in normal weight subjects (*P* < 0.05). These findings were largely independent from age, gender, physical activity and medication use.

When investigating the associations between BMI categories and the risk of wrist fracture using logistic regression models, we found that the crude hazard ratios for overweight and obesity were 2.7 and 5.6 (*P* < 0.001), respectively, when compared to normal weight. However, when accounting for physical function, the hazard ratios of overweight and obese went close to 1. In the final model, falls, lifetime fractures, and Stand‐Sit x5 (s) were significant predictors of fracture, and BMI strata were no longer significant.

**Conclusions:** Although overweight or obesity are not related to mechano‐receptive sensation, they are related to poorer physical function and postural control as well as to poorer perceived confidence and increased dizziness when compared to normal weight. Overweight and obesity are associated with a substantioal increase of wrist fracture risk which can be mainly attributed to poorer physical function in overweight and obese individuals.

**1-34**

**Low blood pressure in heart failure patients with underweight is the predictive factor of mortality and risk factor of frailty**

**Norio Suzuki** ^1^, Keisuke Kida^2^, Shunichi Doi^2^, Chikayuki Ito^2^, Yui Nakayama^1^, Mizuho Kasahara^1^, Shingo Kuwata^1^, Kohei Ashikaga^2^, Manabu Takai^1^, Hisao Matsuda^1^, Koichi Mizuno^1^, Tomoo Harada^2^ and Yoshihiro J. Akashi^2^

^1^ *Division of Cardiology, Department of Internal Medicine, St. Marianna University School of Medicine Yokohama City Seibu Hospital, Yokohama, Japan;* ^2^ *Division of Cardiology, Department of Internal Medicine, St. Marianna University School of Medicine, Kawasaki, Japan*

**Rationale:** Vasodilators are generally used as standard therapy in heart failure. However, patients with malnutrition often develop orthostatic hypotension, and low blood pressure (BP) in underweight patients often decreases activity of daily living.

**Methods:** This study included 135 outpatients with CHF, of which 56 patients with low body mass index (BMI) less than 22 kg/m^2^. All study patients filled in the questionnaire Kihon checklist (KCL) prepared for nursing care prevention in Japan. A patient with 8 points or more was diagnosed as having frailty. Heart failure hospitalization and all‐cause mortality were defined as an event.

**Results:** Overall, the mean age was 76.0 ± 7.4 years old, BMI was 23.0 ± 3.9 kg/m^2^, and left ventricular ejection fraction was 43.6 ± 17.2%. Of the study patients, 54.7% patients were male, 20.7% patients had ischemic heart failure, 46.3% patients had low BP (less than systolic bp 120 mmHg), and 43.7% patients were in the frailty group. In low BMI group, the 1‐year event‐free survival rates were 63.6% and 86.0% in the low BP and another groups (Log‐rank, *P* = 0.001), and the multivariate Cox regression analysis indicated that the hazard ratios was 5.74 in low BP (95% confidence interval, CI; 1.84–22.01, *P* = 0.002). The frailty was set as a target parameter, and the multivariate logistic regression analysis indicated that the odds ratios was 3.89 in low BP (95% CI; 1.27–11.86, *P* = 0.014) in low BMI.

**Conclusions:** Low BP in heart failure patients with underweight might be independent predictive factor for mortality and risk factor for frailty. It was suggested that drug treatment with attention to excessive depression is important in heart failure patients with underweight.

**1-36**

**EWGSOP‐defined sarcopenia stages, bone mineral density, and the prevalence of osteoporosis in Brazilian older woman**

**Ricardo M. Lima**, Rafael R. Lemos, Silvia G.R. Neri and André B. Gadelha

*Faculty of Physical Education, University of Brasília, Brasília, Distrito Federal, Brazil*

**Introduction:** Although there is evidence of an interconnection in the presence of osteoporosis and sarcopenia, little is known about the association between the stages of sarcopenia and osteoporosis. The aim of this study was to assess the association between different stages of sarcopenia, bone mineral density (BMD), and the prevalence of osteoporosis in older women.

**Methods:** Two hundred thirty‐four women (68.3 ± 6.3 years) underwent body composition and BMD measurements using DXA. Quadriceps isokinetic strength was assessed, and the timed up and go test was conducted as a measure of physical function. Sarcopenia stages were categorized according to European Working Group on Sarcopenia in Older People (EWGSOP): nonsarcopenia, presarcopenia, sarcopenia, and severe sarcopenia. The presence of osteoporosis was ensured as a BMD at the hip or spine ≤2.5 standard deviations below the mean of a young‐adult reference population. ANOVA models, chi‐squared tests, and odds ratio were calculated, with significance level set at *P* < 0.05.

**Results:** Osteoporosis prevalence was 15.8%, 19.2%, 35.3%, and 46.2% for nonsarcopenia, presarcopenia, sarcopenia, and severe sarcopenia (x^2^ = 0.017; *P* = 0.002), respectively. Both BMD values and T‐scores were significantly lower among all sarcopenia stages than nonsarcopenic subjects (all *P* < 0.05, η^2^_p_: 0.113 to 0.109). Volunteers classified as severe sarcopenia presented a significantly greater risk for the presence osteoporosis (OR: 3.991; 95% CI: 1.286–12.391). Also, the presence of Sarcopenia (i.e. sarcopenia + severe sarcopenia) was independently associated with a higher risk for osteoporosis (OR: 3.445; 95% CI: 1.521–7.844) when compared to the nonsarcopenia group.

**Conclusions:** These results provide support for the concept that a dose–response relationship exists between sarcopenia stages, BMD, and the presence of Osteoporosis. These findings strengthen the clinical significance of the EWGSOP sarcopenia definition and indicate that severe sarcopenia should be viewed with attention by health professionals.

**1-38**

**Sex difference in frailty in patients with transcatheter aortic valve replacement**

**Mike Saji** ^1^, Ryosuke Higuchi^1^, Kenichi Hagiya^1^, Itaru Takamisawa^1^, Nobuo Iguchi^1^, Jun Shimizu^2^, Shuichiro Takanashi^1^, Tetsuya Tobaru^1^, Morimasa Takayama^1^ and Mitsuaki Isobe^1^

^1^ *Cardiology, Sakakibara Heart Institute, Tokyo, Japan;* ^2^ *Anesthesioliogy, Sakakibara Heart Institute, Tokyo, Japan*

**Introduction:** Variable frailty markers have been developed to guide better patient selection in transcatheter aortic valve replacement (TAVR). However, sex difference in frailty has not been well investigated in this population. This study aimed to investigate the sex difference in frailty and their outcomes in patient with TAVR.

**Methods:** We retrospectively reviewed 155 patients. Short physical‐performance battery (SPPB), Placement of AoRTic TraNscathetER Valve (PARTNER) frailty scale, frailty index, clinical frailty scale, modified Fried scale, and gait speed were calculated. The primary endpoint was all‐cause mortality at 1 year following TAVR.

**Results:** There were 56 men and 99 women in this study. Women were older (85 vs. 82 years, *P* = 0.035) and had smaller body surface area (1.3 vs. 1.5, *P* < 0.001) and aortic valve area (0.60 vs. 0.69 mm^2^, *P* < 0.001) with similar Society of Thoracic Surgeons Scores (7.2 vs. 7.6%, *P* = 0.635) to men. From the standpoint of physical performance in frailty, women were considered to be more frail than men (hand grip strength; 10 vs. 20 kg, *P* < 0.001, gait speed; 0.8 vs. 0.7 m/s, *P* = 0.03), although their scores (not the actual numbers) were not significant (SPPB; 8.1 vs. 8.9, ns, PARTNER frailty scale; 7.1 vs. 6.8, ns, modified Fried scale; 2.4 vs. 2.1, ns). On the other hand, from the standpoint of activity of daily living in frailty, women were also considered more frail than men (frailty index 4.0 vs. 3.1, *P* = 0.05), although simplified classification were not significant (KATZ 5.6 vs. 5.5, ns, clinical frailty scale 4.2 vs. 4.2, ns). Although device success and safety endpoint at 30 days were not different (93 vs. 89%, ns, and 82 vs. 80%, ns, respectively) between them, 1‐year mortality was higher in men (17 vs. 4%, *P* = 0.041).

**Conclusions:** This study suggests that women have greater physiological reserve than men despite advanced age and frailty.

**1-39**

**Prognostic role of muscle, fat, and bone mass in heart failure**

**Masaaki Konishi** ^1^, Eiichi Akiyama^1^, Yasushi Matsuzawa^1^, Ryosuke Sato^1^, Noriaki Iwahashi^1^, Kiyoshi Hibi^1^, Masami Kosuge^1^, Kazuo Kimura^1^ and Kouichi Tamura^2^

^1^ *Division of Cardiology, Yokohama City University Medical Center, Yokohama, Japan;* ^2^ *Department of Medical Science and Cardiorenal Medicine, Yokohama City University Graduate School of Medicine, Yokohama, Japan*

**Background:** There is a paucity of data regarding prognostic role of muscle, fat, and bone mass in patients with heart failure (HF).

**Methods:** We retrospectively analysed 418 patients admitted with a diagnosis of HF [71 ± 13 years, 59% male, and 67% NYHA Class 4 on admission, 54%, 15%, and 31% with reduced left ventricular ejection fraction (LVEF <40%), mid‐range (40% ≤ LVEF <50%), and preserved (≥50%) LVEF, respectively]. Dual‐energy X‐ray absorptiometry was performed at stable state after decongestion therapy. Muscle, fat, and bone mass were indexed by height squared and dichotomized by the median value for each sex. In a same period, 272 non‐HF patients with coronary artery disease were also analysed for comparison.

**Results:** From data of 198 matched pair by age and sex, patients with HF had lower appendicular skeletal mass index (ASMI) than those without HF (7.0 ± 1.3 vs 6.7 ± 1.3 kg/m^2^, *P* = 0.007). Mean ASMI in all HF patients was 6.9 ± 1.2 kg/m^2^ in men and 5.6 ± 0.9 in women, so that 53% of patients had low muscle mass categorized by the Asian Working Group of Sarcopenia. During median follow‐up of 677 days, 159 patients experienced primary outcome defined as death or heart failure hospitalization. Uni‐ and Multivariate Cox regression analysis revealed that lower ASMI [unadjusted HR: 1.85, 95% CI 1.35–2.55, *P* < 0.001; adjusted HR (aHR): 1.47, 95% CI: 1.00–2.17, *P* = 0.048] and bone mass (aHR: 1.48, 95% CI: 1.05–2.10, *P* = 0.027), but not lower fat mass (aHF: 0.84, 95% CI: 0.54–1.31, *P* = 0.45), were associated with elevated risk of primary outcome. Negative impact of lower ASMI was observed for each of outcome components (*P* < 0.0001, *P* = 0.0007, *P* = 0.032, and *P* = 0.0025 by Log‐Rank, for all‐cause death, cardiovascular death, non‐cardiovascular death, and HF re‐hospitalization, respectively).

**Conclusions:** HF was characterized by lower muscle mass in comparison with non‐HF patients. Indices about muscle and bone mass rather than fat mass have prognostic impact in HF.

**1-51**

**Association between PG‐SGA and lean mass determined by computed tomography in patients with cervical cancer**

**Fernanda de Oliveira Pereira**, Izabel Cristina Ramos Cardoso, Mariah Azevedo Aredes and Gabriela Villaça Chaves

*Department of Nutrition and Dietetics, National Cancer Institute José Alencar Gomes da Silva (INCA), Rio de Janeiro, Brazil*

**Introduction:** The most frequent gynaecological tumour is cervical cancer. In clinical practice, Scored Patient‐Generated Subjective Global Assessment (PG‐SGA) appears as a validated subjective assessment tool for cancer patients of low cost, easy, and wide use. On the other hand, Computed Tomography (CT) is a gold standard instrument for determining body composition.

**Methods:** This is a cross‐sectional observational study carried out with women, over 18 years of age, diagnosed with cervical cancer enrolled in a cancer referral hospital in Brazil, who began the treatment between January 2015 and September 2017. Only patients who have PG‐SGA and CT images of the third lumbar vertebra (L3), with maximum interval between them of 45 days, before starting treatment were included. Sociodemographic data and clinical information related to the treatment were collected. The skeletal muscle index (SMI) was used to classify sarcopenia, according to the cut‐off point established for females (≤38.5 cm^2^/m^2^). The data obtained was analysed anonymously with the statistical program Statistical Package for Social Sciences version 22.0. For all statistical analysis, a significance level of 5% has been adopted.

**Results:** The study population consisted of 60 women (age 46.22 ± 11.79 years). The majority of sarcopenic patients were classified as malnourished by PG‐SGA. The patients among with excessive weight by BMI were classified as eutrophic by SMI. Most of patients classified as score 1 in the muscle mass depletion or in the physical examination of PG‐SGA were eutrophic by SMI (95%), and all those who were classified as the worst depletion score (score 4) were sarcopenic.

**Conclusions:** PG‐SGA is shown to be a useful and viable method that has a good association with SMI by CT, being a recommended method of nutritional assessment for patients with cervical cancer.

**1-58**

**Role of volume status on outcomes in chronic haemodialysis patients prone to intradialytic blood pressure changes**

**Su Hyun Kim**, Shin Jung‐Ho and Hwang Jin Ho

*Chung‐Ang University Hospital, Seoul, Korea*

**Background:** Changes in blood pressure frequently occur during the haemodialysis treatment, but their extreme forms, intradialytic hypotension and hypertension, are make difficult keep the treatment. Moreover, recent studies have found that they result in adverse outcomes among chronic haemodialysis patients. Both intradialytic hypotension and hypertension are known to be related with volume status and its change during haemodialysis; however, whether volume status is responsible for their adverse outcomes remains uncertain. Therefore, we evaluated volume status assessed by bioelectrical impedance analysis (BIA), and then, we investigated its influence on cardiovascular event in chronic haemodialysis patients with intradialytic hypotension or hypertension.

**Methods:** In end‐stage renal disease patients on chronic haemodialysis, blood pressure measurements were collected from six haemodialysis sessions before body composition analysis. We defined intradialytic hypotension as a decrease in systolic blood pressure ≥30 mmHg during the treatment in 4/6 treatments and intradialytic hypertension as an increase in systolic blood pressure ≥10 mmHg from pre‐ to post‐haemodialysis in 4/6 treatments. Then, we estimated volume status as the ratio of extracellular water to total body water (ECW/TBW) using a BIA device.

**Results:** A total of 136 chronic haemodialysis patients were followed for 34 (19, 64) months. Of included patients, 34 (24.1%) and 25 (17.7%) were classified into the intradialytic hypotension and hypertension groups, respectively. The ECW/TBW of pre‐haemodialysis and post‐haemodialysis did not differ between the groups (*P* = 0.386 and 0.088). Despite statistical insignificance, post‐haemodialysis ECW/TBW was slightly higher in the intradialytic hypertension group than those in the control and intradialytic hypotension groups (0.38 ± 0.02 vs. 0.37 ± 0.02 vs. 0.37 ± 0.2; *P* = 0.086 and 0.305, respectively). Cardiovascular events occur in 28 cases during the study period, and the incidence differed between the groups (*P* < 0.001). The risk of cardiovascular events was higher in the intradialytic hypotension and hypertension groups than it was in the control group (intradialytic hypotension: HR 5.2, 95% CI 2.1 to 12.8 and intradialytic hypertension: HR 3.3, 95% CI 1.2 to 9.5). Furthermore, the associations between intradialytic blood pressure abnormalities and cardiovascular events persisted after the adjustment by both pre‐ and post‐haemodialysis ECW/TBW (all *P* < 0.05).

**Conclusions:** This study showed that both intradialytic hypotension and hypertension might cause cardiovascular events in chronic haemodialysis patients, and the relationships were independent of volume status. Therefore, further studies for the management of these complications are needed by focusing on other factors related to changes in blood pressure during haemodialysis, such as autonomic system or endothelial function, in addition to efforts to maintain the euvolemic status.

**1-59**

**Muscle mass and its association with frailty in elderly oncologists**

**Maria Jose Molina‐Garrido** ^1^, Maria del Carmen Soriano‐Rodríguez^1^ and Alfonso Muriel^2^

^1^ *Hospital General Virgen de la Luz in Cuenca, Cuenca, Spain;* ^2^ *Unidad de Bioestadística, Hospital Universitario Ramón y Cajal Madrid, Madrid, Spain*

**Objectives:** The main objective of this work is to evaluate which parameters of physiological reserve (including skeletal muscle mass) are associated with frailty, as measured by the VES‐13 questionnaire, in elderly patients with cancer.

**Material and methods:** Prospective observational study of cohorts that included patients ≥70 years of age with cancer, consecutively assessed in a hospital in the central area of Spain.

Variables related to the study population were recorded (age, sex, cultural level, and marital status); with the tumour (type of tumour, tumour stage, receiving or not treatment with chemotherapy, and type of regimen used); physiological reserve parameters of the elderly at different levels: respiratory capacity (expiratory flow measured by a peak‐flow device); renal reserve (creatinine clearance, recorded by the Crockcroft‐Gault formula); cognitive reserve (Pfeiffer questionnaire); muscle reserve (skeletal muscle mass index) and functional reserve (handgrip determined by means of a Jamar manual dynamometer) and speed of walking (a 5‐m stretch). Frailty is defined as a score ≥ 3 in the VES‐13 questionnaire.

By means of binary regression, and later, logistic regression, we determined which of these parameters are correlated with the risk of frailty in the elderly oncology, adjusting for age, ECOG, and sex.

**Results:** A total of 207 patients were included, average age of 78.6 years; male sex (62.3%); 32.7% of patients (*n* = 67) were frail patients. The only physiological reserve variables in the elderly oncology that are associated with frailty are the speed of walking (OR 0.45, 95% CI: 0.008–0.288, *P* = 0.001), the skeletal muscle mass (OR 1.171, 95% CI: 1.046–1.310, *P* = 0.006), and hand grip strength (OR 0.944, 95% CI: 0.904–0.986, *P* = 0.010) (AUC of this model, 0.84, 95% CI: 0.78–0.90).

**Discussion:** The skeletal muscle mass is associated with frailty in measured by the VES‐13 questionnaire.

**1-62**

**Association between handgrip dynamometry (HGD) and health related quality of life (HRQoL) in men with metastatic castrate‐resistant prostate Cancer (mCRPC)**

**Luka Cavka** ^1^, M. Pohar Perme^3^, Nada Rotovnik Kozjek^2^ and Bostjan Seruga^1^

^1^ *Divison of Medical Oncology, Institute of Oncology Ljubljana, Ljubljana, Slovenia;* ^2^ *Department for Clinical nutrition, Institute of Oncology Ljubljana, Ljubljana, Slovenia;* ^3^ *Institute for Biostatistics and Medical Informatics, Faculty of Medicine, University of Ljubljana, Ljubljana, Slovenia*

**Background:** The cornerstone of metastatic prostate cancer treatment represents androgen deprivation therapy (ADT) which could lead to muscle mass loss and consequently impaired functionality, possibly reflected in impaired HRQoL. We aimed to evaluate the association between the functional parameter of nutritional status (HGD) and HRQoL.

**Methods:** We prospectively enrolled men with newly diagnosed mCRPC into this study. At their presentation, we measured handgrip strength by the dynamometer, calculated body mass index (BMI), and obtained phase angle (PA) by the bioimpedance analysis device BodyStat. The HRQoL was evaluated by the Functional Assessment of Cancer Treatment‐Prostate (FACT‐P) questionnaire. The association between HGD and HRQoL was assessed using univariate linear regression; the multivariate model was adjusted for age, duration of ADT, BMI, and PA.

**Results:** We included 109 patients. Patients' median age was 68.1 years (IQR: 68.1–79.3), median duration of ADT 23 months (IQR: 12.7–44.2), median BMI 26.6 kg/m^2^ (IQR: 24.6–30), median PA 4.5° (IQR: 3.9–5.2), median HGD 70 pounds (IQR: 57.5–85.5), and median FACT‐P score 105 points (IQR: 87.5–124.3). In the univariate model, HRQoL score correlated significantly with HGD (*P* < 0.0001, β = 0.47 95% CI [0.24–0.71]; *R* ^2^ = 12.5%), the result remained significant after adjusting for age, ADT, BMI, and PA (*P* < 0.001, β = 0.43 95% CI [0.15–0.72]). In the multivariate model, correlation between PA and HRQoL was also significant after excluding five outliers.

**Conclusions:** In men with newly diagnosed mCRPC, there is an association between nutritional status and HRQoL. This association is only significant when the HGD and PA conveys nutritional status and not traditionally used BMI.

**1-69**

**The effects of experimental cancer and activin receptor ligand blocking on muscle proteome in mice**

**Juha J. Hulmi** ^1,2^, Juulia H. Lautaoja^1^, Jaakko Hentilä^1^, Tuuli A. Nissinen^1^, Fabio Penna^3^, Olli Ritvos^2^, Rabah Soliymani^4^, Marc Baumann^4^ and Maciej Lalowski^4^

^1^ *Neuromuscular Research Center, Biology of Physical Activity, Faculty of Sport and Health Sciences, University of Jyväskylä, Jyväskylä, Finland;* ^2^ *Department of Physiology, Faculty of medicine, University of Helsinki, Helsinki, Finland;* ^3^ *Department of Clinical and Biological Sciences, University of Turin, Turin, Italy;* ^4^ *Meilahti Clinical Proteomics Core Facility, HiLIFE, Faculty of Medicine, Biochemistry and Developmental biology, University of Helsinki, Helsinki, Finland*

**Background and aims:** In a preclinical tumour model blocking of activin receptor (ACVR), ligands improved survival, but only when the treatment was continued after tumour formation (Nissinen *et al*. JCSM 6/2018). The aim of the current study was to examine muscle proteome in these mice.

**Methods:** We injected BALB/c mice with vehicle (control) or C26 cancer cells. Mice were then treated with PBS, or soluble ACVR2B either before the tumour formation (sACVR/b) or with continued treatment also after tumour formation (sACVR/c). Gastrocnemius muscle samples were collected 11 days after the C26 cell injection, lysed, and subjected to nano‐LC‐HD‐MSE analysis (*n* = 7 per group). Differentially expressed proteins quantified with ≥2 unique tryptic peptides, FDR < 0.05, and FC > |1.3|were characterized using Ingenuity Pathway Analysis (IPA) and Western blotting.

**Results:** Over 100 proteins were altered in the skeletal muscle of PBS‐treated tumour‐bearing mice and by the continued (sACVR/c) and the discontinued (sACVR/b) treatments. Overall, principal component analyses and IPA demonstrated that the effects of the treatment termination (i.e. comparison of sACVR/b vs. sACVR/c) on muscle proteome were rather small when compared to the continued group. Pathway Analysis revealed decreased oxidative phosphorylation in the PBS‐treated tumour group, a response which was in part prevented by sACVR. Cancer upregulated the acute phase response pathways predictably through STAT3 in which Serpina3n was the most strongly upregulated protein and especially pronounced in sACVR/b group. The Serpina3n strongly correlated with the loss of body weight from Days 9 to 11 (*r* = −0.82, *P* < 0.001), a time‐point that predicted survival (Cox regression: B = 0.90, *P* < 0.001) in another group of mice (Nissinen *et al*. JCSM 6/2018). The strong correlation in the proteomics signal was validated with Western blotting.

**Conclusions:** Blocking ACVR2B ligands significantly alters muscle proteome in tumour‐bearing mice. Of all the proteins investigated, Serpina3n may be one of the strongest predictors of cachexia and survival.

**1-70**

**Effect of aerobic physical exercise on the skeletal gastrocnemius muscle of spontaneously hypertensive rats**

**Luana Pagan** ^1^, Ricardo L. Damatto^1^, Mariana J. Gomes^1^, Aline R.R. Lima^1^, Felipe Damatto^1^, Marcelo D.M. Cezar^1^, David R.A. Reyes^1^, Ana Angélica H. Fernandez^2^, Marina P. Okoshi^1^ and Katashi Okoshi^1^

^1^ *Botucatu Medical School, Sao Paulo State University, UNESP, Botucatu, Brazil;* ^2^ *Botucatu Institute of Biosciences, Sao Paulo State University, UNESP, Botucatu, Brazil*

**Introduction:** Systemic arterial hypertension promotes cardiac remodelling. With the progression of the disease, ventricular dysfunction occurs and, subsequently, heart failure. Several skeletal muscle alterations occur during this period, such as atrophy, fibrosis, metabolic, enzymatic, and biochemical abnormalities. Currently, physical exercise has been recommended for prevention and recovery of muscle alterations. Therefore, the aim of this study was to evaluate the influence of physical training on oxidative stress, energy metabolism, and respiratory complex in the gastrocnemius muscle of spontaneously hypertensive rats (SHR).

**Methods:** Four experimental groups were used: sedentary (W‐SED, *n* = 19) and trained (W‐EX, *n* = 18) normotensive Wistar rats, and sedentary (SHR‐SED, *n* = 18) and exercised (SHR‐EX, *n* = 22) hypertensive rats. At 13 months old, rats of the exercise groups underwent to treadmill exercise protocol 5 days/week, for 4 months. Echocardiogram was performed to assess *in vivo* cardiac structures and function. At the end of experimental period, white portion of gastrocnemius muscles were obtained for biochemical and histological analysis. Antioxidant enzymes activity, lipid hydroperoxide, energy metabolism, and respiratory complex were evaluated by spectrophotometry. Comparisons between groups were performed by two factors analysis of variance, complemented with the Tukey test (*P* < 0.05).

**Results:** Echocardiogram showed lower left ventricular (LV) wall thickness, LV relative wall thickness, left atrium diameter, and LV relaxation time in SHR‐EX group vs. SHR‐SED. Gastrocnemius lipid hydroperoxide was higher in both hypertensive groups and lower in SHR‐EX vs. SHR‐SED group. Muscle antioxidant enzymes (catalase, superoxide dismutase, and glutathione peroxidase), energy metabolism (phosphofructokinase and citrate synthase), and respiratory complex (Complex I and II) were higher in SHR‐EX vs. SHR‐SED. Lactate dehydrogenase was lower in SHR‐EX vs. SHR‐SED. Both SHR groups presented lower gastrocnemius cross‐sectional area than the respective control groups.

**Conclusion:** Physical exercise attenuates cardiac remodelling, improves oxidative energy metabolism, and decreases oxidative stress in gastrocnemius muscle in spontaneously hypertensive rats.

**Support:** FAPESP; CNPq.

**1-71**

**The effect of exercise intervention depends on cognitive level**

**Mami Fujibayashi** ^1^, Masato Nishiwaki^2^, Noriko Ogawa^1,2^ and Chika Nanayama^2^

^1^ *Setsunan University, Osaka, Japan;* ^2^ *Osaka Institute of Technology, Osaka, Japan*

**Introduction:** A number of studies have demonstrated that exercise training to elderly people has positive effects not only on physical function but also on cognitive function. However, exercise method aimed to prevent cognitive decline is poorly so far is controversial. In the present study, we discuss the effects of fitness classes on physical and cognitive functions by focusing on the individual difference.

**Methods:** 37 healthy elderly volunteers, aged 73.3 ± 5.8, participated in this study. All subjects live in Hirakata city, Osaka prefecture, Japan. They underwent a seated exercise program for 30 min per session, once a week for 8 weeks. Before and after the intervention, body composition measurement, physical fitness ability test, and cognitive function test (Mini‐Mental State Examination, MMSE) were conducted.

**Result:** After the intervention, the results of physical fitness ability test were significantly improved; however, MMSE did not change as a total average value. Next, we divided into two groups [control (≤26, *n* = 29) and cognitive impairment (>26, *n* = 8)] based on the MMSE score and then analysed. As a result, cognitive impairment group significantly improved on the MMSE score. Also, interestingly, physical fitness ability test such as grip strength, 5‐m walking, and long seat flexion value significant improved only in the control group.

**Conclusions:** Although the result that elderly people joining fitness classes only for 8 weeks has positive effects on physical function supports other present studies, division of the data according to the cognitive level revealed that people who are likely to decline in cognitive level did not show significant change in test of physical strength. To these people, long‐term exercise training might be necessary to improve the strength. Despite physical function not improved, the significant improvement in cognitive function is interesting, and this shows the importance of exercise training to elderly people.

**1-72**

**Serum vitamin D is correlated with nutritional status and physical function in maintenance haemodialysis patients**

**Barbara Perez Vogt** ^1^, Sergio Parmezzani Souza^2,3^, João Vitor Neves Santana de Oliveira^1^, Bruno Aparecido Dos Santos^1^, Karoline Ferreira Silva^1^, Thayná Cardoso Diniz^1^, Bianca Barbosa de Farias^1^, Letícia de Lima Oliveira^1^, Luis Alberto Gobbo^2^ and Fabio Santos Lira^3^

^1^ *Health Sciences School, University of Western São Paulo (UNOESTE, Presidente Prudente, Brazil;* ^2^ *Laboratory of Skeletal Muscle Assessment (LABSIM), Physical Education Department, School of Technology and Science, São Paulo State University (UNESP), Presidente Prudente, Brazil;* ^3^ *Exercise and Immunometabolism Research Group, Physical Education Department, School of Technology and Science, São Paulo State University (UNESP), Presidente Prudente, Brazil*

**Introduction:** Patients with chronic kidney disease (CKD) on haemodialysis are at increased risk of protein energy wasting (PEW), leading to impaired exercise capacity, physical disability, reduced quality of life, and increased mortality risk. These patients also exhibit vitamin D deficiency due to conditions, such as aging, obesity, protein loss, and reduced skin production besides impaired kidney function. Vitamin D is associated with functional capacity in several conditions and there is a need for investigation in CKD patients.

**Aim:** The objective of the study was to assess the association of serum vitamin D with PEW and functional capacity in haemodialysis patients.

**Methods:** Cross‐sectional analysis including baseline assessment of patients on maintenance haemodialysis included in a randomized controlled trial that will assess the effect of cholecalciferol supplementation and resistance training on bone mineral metabolism and inflammatory markers, older than 18 years. Participants underwent nutritional status assessments using Malnutrition Inflammation Score (MIS), BMI, and abdominal circumference. Physical function was evaluated by Duke Activity Status Index (DASI). Vitamin D status was defined by serum 25‐hydroxyvitamin D [25(OH)D] and 1,25‐dihydroxyvitamin D [1,25(OH)D].

**Results:** Fourteen patients were included, six males (42.9%), mean age 49 ± 12 years, dialysis vintage 17 (13;63) months. Six patients presented 25(OH)D sufficient levels (≥30 ng/mL) (mean 34 ± 13.8 ng/mL) and three patients presented 1,25(OH)D sufficient levels (≥25 pg/mL) [median 15.3 (10.5;20.6) pg/mL]. There is significant correlation between 25(OH)D and MIS (*r* = 0.632; *P* = 0.037), DASI (*r* = 0.644; *P* = 0.033), and abdominal circumference (*r* = −0.615; *P* = 0.044), and statistical trend with BMI (r = −0,591; *P* = 0.56). Although 25(OH)D and 1,25(OH)D were strongly correlated (*r* = 0.773; *P* = 0.005), correlations of 1,25(OH)D with MIS (*r* = 0.535; *P* = 0.06) and DASI (*r* = 0.258; *P* = 0.373) were not significant.

**Conclusions:** Serum 25(OH)D is correlated with nutritional status and physical function. Further investigation is needed to verify if nutritional or active vitamin D improves those parameters, which may improve quality of life and survival in this population.

**1-73**

**Continuous ICT enabled recording vs. standard functional assessment can reliably indicate physical frailty status in community dwelling older adults**

**Alberto Rainoldi** ^1^, Lorenzo Maria Donini^2^, Luca Carlo Feletti^3^, Gianluca Zia^3,4^ and Susanna Del Signore^4^

^1^ *NeuroMuscularFunction Research group, School of Exercise and Sport Sciences, Department of Medical Sciences, University of Turin, Turin, Italy;* ^2^ *Sapienza University, Rome, Italy;* ^3^ *Caretek srl, Torino, Italy;* ^4^ *BlueCompanion ltd, London, UK*

**Introduction:** Physical Frailty and Sarcopenia (PF&S, Del Signore, Roubenoff, 2017), represents an underestimated health risk among older adults leading to increased morbidity (including falls/injurious falls) and mobility disability. In physically frail older adults, mobility should be carefully assessed in order to prevent further deterioration. In a previous study, the ADAMO watch tested in heathy adults (age >65 years) showed high validity in step detection at slow walking speeds, with an absolute error of 1.5% at 0.8 m/s (Rainoldi *et al*. 2018). In the present study, we analysed the mobility data provided by ADAMO system with the objective to detect a frail/pre‐frail status.

**Methods:** Twenty‐five community‐dwelling older adults (71 ± 6 years; 60% women) worn continuously ADAMO for a week. The mobility index (MI) is a parameter explaining the daily grade of performed physical activity, as: Very Low (VLM), Low (LM), Medium (MM), High (HM), and Very High (VHM) Mobility. Walking ability and physical frailty were estimated using the 400 m walking test and the Tilburg Frailty Indicator (TFI), respectively.

**Results:** Controlling for age and gender, ANCOVA showed that frail and robust participants were different for VLM (frail = 58.8%, robust = 42.0%, *P* < 0.001), LM_MM (frail = 25.5%, robust = 33.8%, *P* = 0.008), and HM_VHM (frail = 15.7%, robust = 24.2%, *P* = 0.035). Using cluster analysis, participants were divided into two groups, with higher or lower mobility. Age and gender controlled linear regression showed that the MI clusters were associated with total (β = 0.571, *P* = 0.002) and physical frailty (β = 0.381, *P* = 0.031); and the 400mWT was associated with total (β = 0.404, *P* = 0.043) and physical frailty (β = 0.668, *P* = 0.002).

**Conclusions:** ADAMO wristwatch demontrated being a reliable mobility tracking system to record, non‐intrusively, continous data on mobility levels closely related to other objective (400mWT) or subjective indicators (TFI) of the physical frailty status. As a next step, longitudinal studies in homogeneous populations suffering from Physical Frailty and Sarcopenia should determine ADAMO MI suitability as an endpoint for clinical trials.

**1-74**

**Comparison of muscle size, muscle composition, and physical function between healthy and physically disabled older adults**

**Yuya Watanabe** ^1^, Shogo Gyoba^1^, Emi Yamagata^2^, Daiki Hara^1^, Yasuaki Kamada^1^, Junpei Tsutsumi^1^ and Shushi Fukuhara^1^

^1^ *Faculty of Health and Sports Science, Doshisha University, Kyoto, Japan;* ^2^ *Faculty of Nursing, Doshisha Women's College of Liberal Arts, Kyoto, Japan*

**Introduction:** Loss of skeletal muscle mass is a major well‐known change associated with aging. It is reported that qualitative changes such as increased inter or intramuscular adipose and connective tissue in skeletal muscle with aging. These changes in muscle composition are associated with decreased motor function and/or insulin resistance. Muscle quality is considered to be related to frailty of the older individuals. Recently, echo intensity (EI) of skeletal muscle obtained by ultrasonography has been considered to reflect muscle quality. This study aimed to compare muscle size, muscle quality, and physical function between healthy and physically disabled older adults.

**Methods:** A total of 98 older adults participated in this study: healthy group (15 males and 23 females; 65–90 years) and disable group (25 males and 35 females; 66–94 years). All participants in physically disabled group were individuals requiring long‐term care or support. Muscle thickness (MT) and EI of the anterior compartment of the right thigh were evaluated using ultrasonography imaging method. Physical functions such as knee extension strength and walking speed were also measured.

**Results:** In both sexes, healthy group had significantly higher MT (*P* < 0.01) and lower EI (*P* < 0.05) than disabled group. Knee extension strength and walking speed in healthy group were significantly higher than those in disabled group (*P* < 0.001). When multiple regression analysis was performed, after adjusting age, sex, group, and body mass index, MT, EI, and group were still significantly correlated with knee extension strength (*P* < 0.01). However, EI was not significantly correlated with maximal walking speed (*P* = 0.629).

**Conclusions:** This study indicate that the quality in addition to the quantity of the lower limb muscle affects the physical functional decline in older people. However, the impact of muscle quality is thought to be less than that of muscle size.

**1-75**

**Phase angle is associated with physical performance in patients on peritoneal dialysis**

Maryanne Zilli C. Silva^1^, Vanessa Mota da Silva^2^, Nayrana Soares do Carmo Reis^1^, **Barbara Perez Vogt** ^3^ and Jacqueline Costa Teixeira Caramori^4^

^1^ *Post‐graduation in Pathophysiology in Internal Medicine, São Paulo State University (UNESP), Botucatu, Brazil;* ^2^ *Under Graduation in Nutrition, Biosciences Institute, São Paulo State University (UNESP), Botucatu, Brazil;* ^3^ *Health Sciences School, University of Western São Paulo (UNOESTE), Presidente Prudente, Brazil;* ^4^ *Department of Internal Medicine, Botucatu Medical School, São Paulo State University (UNESP), Botucatu, Brazil*

**Introduction:** Sarcopenia is characterized by reduced muscle mass and physical function or performance. Physical performance reduction is associated with poor outcomes in patients with chronic kidney disease (CKD) on dialysis. Phase angle (PA) reflects nutritional status, and it is also an important prognostic indicator, besides it is a non‐invasive and objective assessment. However, there are no studies associating PA with physical performance in PD patients.

**Aim:** The objective of the study was to evaluate the association between phase angle (AF) with functional capacity in patients with CKD on peritoneal dialysis (PD).

**Methods:** Cross‐sectional study, which enrolled PD prevalent patients, older than 18 years. AF evaluation was performed by bioelectrical impedance and functional capacity evaluated by Short Physical Performance Battery (SPPB), which includes balance, gait speed, and sit‐to‐stand tests. For statistical analysis, Spearman's correlation and Multiple Linear regression were used. The level of significance was set at *P* < 0.05.

**Results:** Fifty patients in PD, 52% women, mean age 55.7 ± 16.2 years, median time of therapy 9.5 (5–18) months, BMI of 26 ± 4.5 kg/m^2^ and AF 6.06 ± 0.96. Regarding SPPB, 48% of the sample presented high performance, 46% intermediate performance, and 6% low performance. There is a significant correlation of PA with SPPB score (*r* = 0.481, *P* = 0.000), gait speed test (*r* = 0.479, *P* < 0.001), and balance and sit‐to‐stand tests (*r* = 0.294, *P* = 0.038, *r* = 0.374, *P* = 0.007, respectively). In regression model (*R* ^2^ = 0.379), the variables balance (OR = 0.333, IC 0.548–0.2793, *P* = 0.007) and gait speed (OR = 0.492, IC 0.247–0.803, *P* = 0.000) were associated with PA.

**Conclusions:** There is significant association of SPPB score with PA, as well with the isolated variables included in SPPB. Since PA is associated also with nutritional status, regular monitoring of PA provides an important assessment, which may improve outcomes in patients in PD.

**1-77**

**Depressed heart rate variability associated with poor functional outcome after early post‐stroke rehabilitation**

**Nadja Scherbakov** ^1,2^, Anush Barkhudaryan^3^, Nicole Ebner^4^, Stephan von Haehling^4^, Stefan D. Anker^5,6^, Michael Joebges^7^ and Wolfram Doehner^1,2,5,6^

^1^ *Center for Stroke Research Berlin, Charite Universitätsmedizin Berlin, Berlin, Germany;* ^2^ *German Centre for Cardiovascular Research (DZHK), Partner Site Berlin, Berlin, Germany;* ^3^ *Clinic of General and Invasive Cardiology, University Clinical Hospital № 1, Yerevan, Armenia;* ^4^ *Department of Cardiology and Pneumology, University of Göttingen, Göttingen, Germany;* ^5^ *Department of Cardiology, Charite University Medical School, Berlin, Germany;* ^6^ *Berlin‐Brandenburg Center for Regenerative Therapies (BCRT), Charité‐Universitätsmedizin Berlin, Berlin, Germany;* ^7^ *Department of Neurology, Brandenburgklinik Bernau, Bernau bei Berlin, Germany*

**Background:** Impaired autonomic nervous system regulation is frequently observed in patients with stroke. Previously, an association of increased sympathetic activity with unfavourable functional outcome has been shown in post‐stroke patients. The aim of the present study was to evaluate the impact of heart rate variability (HRV) assessed by the time‐domain method on functional outcome in patients with subacute stroke.

**Methods:** 105 consecutive patients (70 ± 11 years, BMI 27.0 ± 5.4 kg/m^2^, 63% males) with ischaemic or hemorrhagic (15% of patients) stroke were studied during the early post‐stroke rehabilitation (Brandenburgklinik, Bernau, Germany). All patients underwent 24‐h Holter‐monitoring at admission. Study examinations were performed at begin of rehabilitation (23 ± 17 days post‐stroke, p.s.) and discharge (50 ± 18 days p.s.). The functional status was assessed by Barthel Index (BI), modified Rankin scale (mRS), and Rivermead Motor Assessment (RMA). Depressed HRV was defined by HRV‐TI ≤20 and SDNN <100 ms. Cumulative functional disability was defined by the cumulative presence of mRS ≥ 4 points, BI ≤ 70 and RMA ≤ 5 at discharge.

**Results:** In total, 20 patients (19%) were found with depressed HRV at admission to the early post‐stroke rehabilitation. The functional status (mRS, RMA, and BI) after stroke at the beginning of the rehabilitation did not differ between patients with normal HRV and with depressed HRV (mRS and BI, both *P* > 0.08; RMA, *P* = 0.2) At discharge, patients with depressed HRV showed the lowest functional status according to the BI (60 ± 23 vs. 76 ± 20, *P* < 0.0001), mRS (3.7 ± 0.6 vs. 3.0 ± 1.0, *P* < 0.001), and RMA (6.4 ± 2.3 vs. 7.4 ± 2.2, *P* < 0.05, all analysed by ANCOVA adjusted for baseline) as compared to the patients with normal HRV. After adjustment for body mass index, age, sex, β‐blocker, Ca^2+^‐antagonists and the presence of diabetes mellitus, cumulative functional disability was independently associated with depressed HRV (OR 3.87 [95% CI 1.29–11.59], *P* = 0.016).

**Conclusions:** Increased sympathetic nervous system activity (depressed HRV) in patients with stroke was associated with the worst functional outcome after the early post‐stroke rehabilitation.

**1-78**

**Serum C1q level is a novel biomarker of cardiovascular diseases risk**

**Natsuki Hasegawa** ^1^, Naoki Horii^1,2^, Shumpei Fujie^2,3^, Masataka Uchida^1^, Kiyoshi Sanada^1^, Takafumi Hamaoka^4^ and Motoyuki Iemitsu^1^

^1^ *Ritsumeikan University, Kyoto, Japan;* ^2^ *Research Fellow of Japan Society for the Promotion of Science, Japan;* ^3^ *University of Tsukuba, Tsukuba, Japan;* ^4^ *Tokyo Medical University, Tokyo, Japan*

**Introduction:** Sarcopenia induces an elevation of cardiovascular disease risks, such as arterial stiffness index. Recently, C1q has been identified as a novel myokine, and the expression of muscle C1q mRNA increases with aging. Furthermore, we revealed that aging‐induced muscle C1q secretion leads to muscle fibrosis and atrophy via activation of Wnt/β‐catenin signalling. Additionally, another study has been shown that, *in vivo* and *in vitro* studies, C1q induces proliferation of vascular smooth muscle cells. However, it is unclear whether the aging‐induced secretion of myokine, C1q, TNF‐alpha, and IL‐6 levels were associated with cardiovascular disease risk in humans. Therefore, this study aimed to clarify whether serum C1q, TNF‐alpha, and IL‐6 levels are associated with aging‐induced increase in cardiovascular disease risk.

**Methods:** One‐hundred twenty‐seven subjects (18–81 years, male: *n* = 67, female: *n* = 60) participated in this study. Subjects were divided into two groups; young (<40 year) and middle‐aged and older (≥40 year) groups. Serum C1q, TNF‐alpha, and IL‐6 levels were assessed by ELISA. Arterial stiffness index, as an index of cardiovascular disease risk, was estimated by using carotid‐femoral pulse wave velocity (cfPWV). MRI was used to determine muscle cross‐sectional area (CSA) of quadriceps and hamstrings.

**Results:** Muscle CSA was negatively correlated with cfPWV (*r* = −0.41, *P* < 0.05). Serum C1q, TNF‐alpha and IL‐6 levels, and cfPWV were significantly higher in middle‐aged and older group as compared with young group (respectively *P* < 0.05). Furthermore, serum C1q level was negatively correlated with muscle CSA (*r* = −0.25, *P* < 0.05), whereas no significant correlation was observed between serum TNF‐alpha or IL‐6 and muscle CSA. Additionally, the serum C1q level was positively correlated with cfPWV (*r* = 0.47, *P* < 0.05). After adjusting for 11 confounders, the association between serum C1q level and cfPWV remained statistically significant (ß = 0.24, *P* < 0.05).

**Conclusions:** These results suggest that serum C1q level may be a novel biomarker of the elevation of arterial stiffness with age.

**1-79**

**Determinants of bone status in men with heart failure: results from the studies investigating co‐morbidities aggravating heart failure (SICA‐HF)**

**Goran Loncar** ^1,2,3^, Tania Garfias Macedo^3^, Nicole Ebner^3,4^, Miroslava Valentova^3,4^, Anja Sandek^3,4^, Breno Godoy^3,4^, Mirela Vatic^5^, Amir Emami^3,4^, Ruben Evertz^3,4^, Mitja Lainscak^6^, Wolfram Doehner^7,8,9^, Stefan D. Anker^7,8,10^ and Stephan von Haehling^3,4^

^1^ *Institute for cardiovascular disease Dedinje, Belgrade, Serbia;* ^2^ *Faculty of medicine, University of Belgrade, Belgrade, Serbia;* ^3^ *Department of Cardiology and Pneumology, University Medical Center Goettingen, Georg‐August University, Goettingen, Germany;* ^4^ *Partner Site Goettingen, DZHK (German Centre for Cardiovascular Research), Goettingen, Germany;* ^5^ *Cardiovascular Science Program, Medical University of Goettingen (UMG), Goettingen, Germany;* ^6^ *Department of Internal Medicine, General Hospital MurskaSobota and Faculty of Medicine, University of Ljubljana, Ljubljana, Slovenia;* ^7^ *Division of Cardiology and Metabolism‐Heart Failure, Cachexia & Sarcopenia, Department of Cardiology (CVK), Charité University Medical Center Berlin, Berlin, Germany;* ^8^ *Berlin‐Brandenburg Center for Regenerative Therapies (BCRT), Charité University Medical Center Berlin, Berlin, Germany;* ^9^ *Center for Stroke Research Berlin (CSB), Charité‐Universitätsmedizin Berlin, Berlin, Germany;* ^10^ *DZHK (German Centre for Cardiovascular Research), Partner Site Berlin, Berlin, Germany*

**Background:** Hear failure (HF) and osteoporosis are highly prevalent aging‐related diseases that exact a huge impact on society. The most devastating complication of osteoporosis is hip fracture, which is associated with disability and high mortality risk. We aimed to assess the determinants of hip bone mineral density (hip BMD) in men with HF.

**Methods:** One hundred ninety‐seven males with HF and 25 healthy controls, aged over 40 years old, were included. Bone status and body composition were measured by DEXA method at baseline and at 30 months, as were spiroergometry and blood analysis.

**Results:** HF patients were older and had higher body mass index than controls (68 ± 10 vs. 63 ± 11 years, 29 ± 5 vs. 25 ± 3 kg/m^2^, *P* = 0.012 and *P* = 0.001, respectively). Lean mass was similar between groups (58 082 ± 8911 vs. 57 122 ± 7754 g, *P* = 0.61), while fat mass was increased in HF group (27 721 ± 9462 vs. 18 936 ± 6926 g, *P* < 0.0001). After adjustment for body weight, hip BMD was decreased in HF subjects (0.0135 ± 0.0025 vs. 0.0146 ± 0.0018 g/cm^2^, *P* = 0.04). Hip BMD was lower in NYHA III vs. NYHA I/II (*P* = 0.03). Patients with decreased hip BMD (below median value) compared to those with normal hip BMD (above median value) were older (70 ± 10 vs. 66 ± 10 years, *P* = 0.01) and more symptomatic (NYHA class 2.4 ± 0.6 vs. 2.2 ± 0.6, *P* = 0.02), while left ventricular ejection fraction and renal function were similar (*P* > 0.05 for both). Patients with decreased hip BMD presented with lower lean mass (55 746 ± 8863 vs. 60 491 ± 8342 g, *P* < 0.0001), while fat mass was similar compared to the patients with normal hip BMD (26 752 ± 10 310 vs. 28 721 ± 8437 g, *P* = 0.15). Physical performance was inferior in patients with decreased hip BMD (absolute peak VO2: 1419 ± 508 vs. 1646 ± 445 mL/min, *P* = 0.003). Proinflammatory milieu, expressed by cytokines levels, was emphasized in HF patients with reduced hip BMD compared to normal hip BMD [for TNF‐α 205 (164) vs. 182 (97) pg/mL, *P* = 0.025; for IL‐1β 9.95 (6.96) vs. 9.02 (4.23) pg/mL, *P* = 0.024; for IL‐6 40.16 (30.43) vs. 38.14 (18.62) pg/mL, *P* = 0.032]. Decreased hip BMD was independently predicted by lower absolute peak VO2 and increased TNF‐α (*P* < 0.05 for both). Hip BMD significantly decreased after 30 months of follow‐up in males with HF (1.179 ± 0.132 vs. 1.170 ± 0.142, *P* = 0.02).

**Conclusions:** In men with HF, impaired exercise capacity and proinflammatory milieu independently correlated with reduced hip BMD. HF patients with decreased exercise capacity and/or higher inflammatory markers should be advised to perform bone assessment in order to prevent potentially fatal hip fracture.

**1-80**

**Duration of reproductive span was significantly associated with the risk of sarcopenia in Korean women**

**Howhwa Chang**, Yujeong Hwang and Eunsuk Cho

*Department of Family Medicine, Yonsei University Wonju College of Medicine, Wonju, South Korea*

**Background:** Sarcopenia is the age‐related loss of skeletal muscle mass and strength which has been highlighted since the decreased muscle mass is prevalent in elderly and demonstrated to be associated with frailty, chronic disease, and mortality in elderly people. Menarche and menopause are two indicators of the reproductive history of women; however, their associations with sarcopenia have not been researched. Therefore, the present study will be to examine association between duration of reproductive span and Sarcopenia.

**Methods:** We used data obtained from the Korea National Health and Nutrition Examination Surveys, which is a nation‐wide cross‐sectional survey. Dual‐energy X‐ray absorptiometry was used to measure body composition. We defined subjects whose muscle mass, especially the weight‐adjusted appendicular skeletal muscle mass was 1 standard deviation below the mean of the young reference group as sarcopenia. Reproductive spans were obtained by calculating durational difference years of menarche and menopause. Furthermore, we split the reproductive span according to tertile. Binary logistic regression analysis was performed to select confounders for sarcopenia. Multivariate logistic regression analysis was used to analyse association between reproductive years and sarcopenia after adjusting confounders. *P* < 0.05 was considered statistically significant.

**Results:** In total, 3963 women were included in this study, 803 were subjects with sarcopenia (20.3%, 803/3963). After adjusting for potential confounders, we found the decreased risk of sarcopenia with longer reproductive span [Tertile 1 = 1 (reference); Tertile 2, odds ratio (OR) = 0.738, 95% confidence interval (CI) = 0.733–0.744; Tertile 3, OR = 0.643, 95% CI = 0.636–0.650].

**Conclusions:** Longer reproductive spans were significantly associated with a decreased prevalence of Sarcopenia.

**1-81**

**Association of nutritional status with muscular function, functional capacity, and quality of life in maintenance haemodialysis patients**

João Marcos Soares Reis^1^, Leticia Salmazzo Alves^1^, Camila Zanini Gimenes Freitas^1^, Simone Cassia Casadei Buchalla^1^, Luciana Kelly Camargos Batista^2^ and **Barbara Perez Vogt** ^1^

^1^ *Health Sciences School, University of Western São Paulo, Presidente Prudente, Brazil;* ^2^ *Presidente Prudente Medical School, University of Western São Paulo, Presidente Prudente, Brazil*

Patients with chronic kidney disease (CKD) on haemodialysis usually develop protein‐energy wasting, decrease in functional capacity and quality of life, increasing sedentary lifestyle, aggravating the situation mass and muscle function loss, chronic systemic inflammation and cardiovascular disease. This study aimed to evaluate the association of nutritional status with muscle function, functional capacity, and quality of life of haemodialysis patients.

**Methodology:** Cross‐sectional study that included patients with CKD on maintenance haemodialysis for at least 3 months, with more than 18 years old. Participants were submitted to a single evaluation of nutritional status, quality of life, muscular function, and functional capacity. Participants were characterized by demographic, clinical, and laboratory data. Malnutrition Inflammation Score (MIS) was used to assess nutritional status. Handgrip strength (HGS) was performed to evaluate muscle function and functional capacity was evaluated by Short Physical Performance Battery (SPPB). Quality of life was assessed by the SF‐36 Quality of Life Questionnaire. To evaluate the patient's physical activity level, International Physical Activity Questionnaire (IPAQ) was used.

**Results:** Seventy‐seven patients were enrolled, 64.9% male, mean age 55 ± 14 years, dialysis vintage 23 (8;72) months, MIS median 5 (3;7), and SPPB median 8 (6;10). Positive correlations between MIS and dialysis vintage and negative correlations between MIS and BMI, creatinine, iron, albumin, total cholesterol, triglycerides, handgrip strength, SPPB, and SF‐36 domains of functional capacity, vitality, and general health were found. Physical activity level was not correlated with MIS. Multiple analysis resulted in a statistically significant model (*R* ^2^ = 0.402; *P* < 0,001) with association of HGS, functional capacity (assessed by SF‐36) and age with MIS.

**Conclusions:** There is an association between nutritional status of patients in maintenance haemodialysis with muscle function, assessed by HGS and functional capacity, assessed by SF‐36.

**Acknowledgements:** This work has been supported by São Paulo Research Foundation (FAPESP) grants 2017/13235‐7 and 2017/13187‐2.

**1-82**

**Muscle function correlates positively with serum creatinine and negatively with CD4 cells in patients on maintenance haemodialysis**

Fabio Santos Lira^1^, Sergio Parmezzani Souza^1,2^, Luis Alberto Gobbo^2^ and **Barbara Perez Vogt** ^3^

^1^ *Exercise and Immunometabolism Research Group, Physical Education Department, School of Technology and Science, São Paulo State University (UNESP), Presidente Prudente, Brazil;* ^2^ *Laboratory of Skeletal Muscle Assessment (LABSIM), Physical Education Department, School of Technology and Science, São Paulo State University (UNESP), Presidente Prudente, Brazil;* ^3^ *Health Sciences School, University of Western São Paulo (UNOESTE), Presidente Prudente, Brazil*

**Purpose:** Skeletal muscle mass and function are impaired in chronic kidney disease (CKD), and it can lead to metabolic disruption, such as dyslipidemia and insulin resistance. Muscle mass is related with serum creatinine in CKD population and can be used as a reliable muscle mass biomarker. Another factor associated with metabolic disruption is immunological system. Changes in percentage of CD4+ and CD8+ T cells may indicate that defence against intracellular pathogens is suppressed. Infection is one of the main causes of mortality in haemodialysis. Therefore, we aimed to evaluate the association of muscle function and metabolic and immunological profile in maintenance haemodialysis patients.

**Methods:** Cross‐sectional analysis including baseline assessment of patients with CKD on haemodialysis included in a randomized controlled trial that will assess the effect of cholecalciferol supplementation and resistance training on bone mineral metabolism and inflammatory markers. Patients were older than 18 years on haemodialysis for at least 3 months. Participants underwent muscle function evaluation using handgrip strength. Metabolic profile (total cholesterol, HDL‐c, LDL‐c, triglycerides, glucose, and creatinine) was determined by colorimetric method. Percentage of CD4+ (%CD4+) and CD8+ (%CD8+) were determined using flow cytometry.

**Results:** Fourteen patients were included, six males (42,9%), mean age 49 ± 12 years, dialysis vintage 17 (13;63) months. Correlations between muscle function and serum creatinine (*r* = 0.692, *P* = 0.009) and %CD4+ cells (*r* = −0.564, *P* = 0.045) were found. There was no significant correlation between serum creatinine and the analysed variables. Triglycerides correlates with %CD4+ (*r* = 0.708; *P* = 0.005), %CD8+ (*r* = 0.537; *P* = 0.048), and CD4+/CD8+ (*r* = 0.586; *P* = 0.028).

**Conclusions:** Muscle function is positively correlated with serum creatinine and negatively with %CD4+ cells. Triglycerides exhibited close correlation with immune cells showing immunometabolic interaction. Decrease in peripheral %CD4+ cells can, at least in part, contribute for immunosuppression condition. Further investigation is needed to verify the interaction between muscle quality and immunological response in patients on haemodialysis.

**1-83**

**A study protocol on physiopathology of neuromuscular function for multi‐comorbidity patients with chronic kidney disease during pre‐dialysis phase**

**Antoine Chatrenet** ^1,2^, Antioco Fois^2^, Durand Sylvain^1^, Beaune Bruno^1^, Jadeau Christelle^3^ and Giorgina Barbara Piccoli^2,4^

^1^ *Laboratoire Motricité, Interactions, Performance, Université du Mans, Le Mans, France;* ^2^ *Néphrologie, Centre Hospitalier du Mans, Le Mans, France;* ^3^ *Centre de Recherche Clinique, Centre Hospitalier du Mans, Le Mans, France;* ^4^ *Dipartimento di Scienze Cliniche e Biologiche, Università di Torino, Torino, Italy*

**Introduction:** Chronic Kidney Disease (CKD) is a worldwide emerging disease that induces progressive and systemic damages with glomerular filtration rate (GFR) decline. Many metabolic derangements appear since the early CKD stages, and their prevalence and importance grown with deterioration of GFR. During the ‘pre‐dialysis’ phase, muscle wasting is multifactorial. Along with inhibition of protein synthesis and increase protein catabolism, more complex phenomena are probably in cause, impacting muscular function rather than a quantitative reduction of the lean mass. Indeed, pre‐dialysis CKD patients are not universally considered as patients with neuromuscular deficiency, but profound fatigue is commonly reported. This study aims to investigate in a high comorbidity cohort of pre‐dialysis patients, the link between reported fatigue rate and neuromuscular capacities, specifically through neuromuscular fatigue (NmF) analysis.

**Methods:** It is a prospective, observational, and non‐randomized study. Inclusion criteria: All the adult patients with CKD 3B – 5 followed in the Unit for the advanced CKD (*n* = 130) will be offered the possibility of performing this evaluation (free of charge). Exclusion criteria: Paediatric and pregnant patients and those who refuse.

Anthropometric, biochemical, and kidney function data will be collected, functional capacity tests like to Timed up and Go (TUG), 6 min walking test (6WT), and specific force analysis during exercise fatigue induced will be realized. Force analysis will collect time to maximal contraction, maximal force, critical force (i.e. maximal isometric force that a muscle can maintain ‘for a very long time without fatigue’), and electromyography analysis will allow interpolate electromyography fatigue threshold with deVries method (1984).

**Results:** Approximately 100–130 patients with multiple comorbidities are expected to be enrolled in this study. Patients will be stratified according to the main deficit, and specific training programs will be designed.

**Conclusions:** The study of neuromuscular deficits could guide specific interventions to enhance muscular efficiency, well‐being, and to potentially improve fatigue.

**1-84**

**Objective measurement of muscle strength**

**Oldřich Vyšata**, Zdeněk Zadák and Martin Vališ

*University Hospital Hradec Králové, Hradec Králové, Czech Republic*

**Introduction:** Muscle force is usually measured by uniformly trained physical therapists. The most widespread methods of measuring muscle strength require patient cooperation. To measure progress of muscle weakness or treatment effect on strength different methods have been used, including manual muscle testing (MMT) and maximal voluntary isometric contraction (MVIC) megascores. Coefficients of variation for single muscle were for both methods in clinical studies 3.5–7.5. In this pilot study, muscle strength is tested objectively by stimulation of the muscle at the motor point.

**Methods:** Muscle contraction of the M. tibialis anterior was induced by increasing electric current intensity stimulation in the motor point of the muscle. The air pressure was measured in the cuff located on the instep of the foot fixed in a solid plunger. Muscle stimulation was terminated at a constant force of muscle contractions with increasing intensity of stimulation. Ten measurements were performed on 12 healthy volunteers over a month. Coefficient of variation was used to estimate intraindividual variability.

**Results:** The dependence of the muscle contraction force measured by the pressure in the cuff on the electrical current intensity is nonlinear. During muscle stimulation at the motor point, the discomfort is less than that of the repetitive stimulation of the mixed nerve, in which sensory fibres are stimulated at the same time. Coefficient of variation was 3.56.

**Conclusions:** The proposed method of objective measurement of muscle strength by repetitive stimulation at the motor point is highly reproducible. It does not require patient collaboration. It requires minimal training of medical technician. The only significant source of variance in the subject being investigated is the placement of the stimulation and reference electrodes above the motor point.

**2-10**

**New insights into muscle atrophy in a model of chronic sciatic nerve constriction**

**Vincenzo Musolino** ^1,2^, Francesca Bosco^1,2^, Saverio Nucera^1,2^, Federica Scarano^1,2^, Miriam Scicchitano^1,2^, Cristina Carresi^1,2^, Stefano Ruga^1,2^, Maria Caterina Zito^1,2^, Micaela Gliozzi^1,2^ and Vincenzo Mollace^1,2,3^

^1^ *Institute of Research for Food Safety and Health (IRC‐FSH), University of Catanzaro “Magna Graecia”, Catanzaro, Italy;* ^2^ *NUTRAMED S.c.a.r.l., Roccelletta di Borgia, Catanzaro, Italy;* ^3^ *IRCCS San Raffaele Pisana, Rome, Italy*

**Introduction:** Chronic constriction injury (CCI) of the rat sciatic nerve is a used model for research of neuropathic pain, but little is known about musculoskeletal changes associated with this injury. We investigated the modulation of catabolic pathways, in skeletal muscle, using a model of atrophy induced by chronic sciatic nerve constriction.

**Methods:** Rats were divided into CCI and naive groups. In CCI group, sciatic nerve of right hind limb (ipsilateral) was exposed and constricted. Left hind limb (controlateral) underwent no surgical procedures. The naive group rats did not undergo surgery. Body weight and composition were measured before the injury, and then after 28 days, rats were anaesthetised, the ipsilateral and controlateral skeletal muscles dissected, and weighed. Cross sectional area (CSA) was visualized by haematoxylin and eosin staining. Catabolic signalling was assessed in gastrocnemius muscle (GC).

**Results:** At 28 days post‐sciatic nerve ligation, the CCI group had a body weight and body composition compared to naive animals. Muscle weight as well as cross sectional area (CSA) of muscle fibres decreased in ispilateral garstrocnemius compared to controlateral side. In the ipsilateral GC of the CCI group, western blotting showed an up regulation of the catabolic regulators (Beclin‐1, p62, TRAF6, and LC3), whereas atrogin‐1 was down regulated. Moreover, Pax‐7 was upregulated. Interestingly, Neutrophil gelatinase‐associated lipocalin (NGAL) protein expression was upregulated in the injured hind limb compared to the contralateral side in CCI group.

**Conclusions:** CCI led to atrophy driven by an increase in autophagic markers. The ubiquitin E3‐ligases atrogin‐1 is decreased, suggesting that its involvement could occur at an earlier time point. Higher Pax7 expression in injured GC was observed, indicating that sciatic nerve damage associates with an expansion of satellite cells in atrophic muscle. Levels of NGAL, which is also upregulated in heart failure, was upregulated in muscle atrophy induced by chronic sciatic nerve constriction, suggesting a novel role of NGAL in muscle wasting.

**2-12**

**Integrative transcriptome and miRNAome analysis reveals extracellular matrix remodelling during muscle atrophy in cancer cachexia**

**Geysson Javier Fernandez**, Juarez Henrique Ferreira, Ivan José Vechetti‐Júnior, Leonardo Nazario de Moraes, Paula Paccielli Freire, Sarah Santiloni Cury, Maeli Dal Pai‐Silva and Robson Francisco Carvalho

*Department of Morphology, Institute of Biosciences, São Paulo State University (UNESP), Botucatu, SP, Brazil*

Cachexia is a complex metabolic syndrome characterized by loss of skeletal muscle, leading to a significant weight loss that impacts patient morbidity and mortality. Given the complexity of gene regulatory networks that control gene expression, our objective was to perform an integrative mRNA and miRNA profiling to identify genetic programs that capture essential mechanistic details promoting muscle atrophy in cancer cachexia. C57BL/6 mice were subcutaneously injected with Lewis Lung Carcinoma cells (LLC) or PBS (Control), and the main features of cancer cachexia were characterized after 23 days. Genome‐wide gene expression profiles of mRNA and miRNA were performed by using RNA‐sequencing in tibialis anterior (TA) muscle. Extracellular matrix (ECM) remodelling was quantified by western blotting and Picrosirius staining in TA. *In vitro* experiments were performed using C2C12 myotubes. LLC mice reduced body weight, presented muscle and fat tissue wasting, and their tumour size negatively correlated with body and TA weights. Additionally, we found 1008 differential expressed mRNAs (487 up‐regulated and 521 down‐regulated) and 18 miRNAs (13 up‐regulated and 5 down‐regulated). Our analysis suggests the activation of transcriptions factors contributing to muscle wasting such as NF‐κB, Stat, AP‐1, and FoxO. Moreover, we identified potential posttranscriptional regulation by miRNAs of ECM organization and secretome components, such as collagens and osteoglycin. C2C12 myotubes treated with TNF‐α and IFN‐γ further validated the effects mediated by inflammation on ECM and secretome miRNAs‐target transcripts. Finally, functional siRNA experiment in C2C12 myotubes confirmed that osteoglycin knockdown induces myotubes atrophy. Our results identify a set of signalling pathways potentially regulated by miRNAs that may contribute to muscle atrophy in cancer cachexia. Moreover, cancer cachexia and the inflammatory cytokines TNF‐α and IFN‐γ induce changes in muscle ECM and secretome components such as osteoglycin, which is associated with muscle cell atrophy.

Grant #14/13941‐0, São Paulo Research Foundation (FAPESP).

**2-13**

**β‐Hydroxy‐β‐methylbutyrate supplementation inhibits pancreatic tumour cell growth and preserves muscle mass**

**Stephen D. Hursting** ^1,2,3^, Kristyn Liu^3^ and Michael Coleman^1^

^1^ *Department of Nutrition, University of North Carolina, Chapel Hill, NC, USA;* ^2^ *Lineberger Comprehensive Cancer Center, University of North Carolina, Chapel Hill, NC, USA;* ^3^ *Department of Nutritional Sciences, University of Texas, Austin, TX, USA*

**Introduction:** Cachexia, a complex catabolic state, frequently accompanies pancreatic ductal adenocarcinoma (PDAC), the fourth leading cause of cancer‐related death in the US and the seventh leading cause of cancer death worldwide. We previously showed in a murine PDAC model that leucine supplementation improves muscle protein synthesis but also enhances PDAC growth through activation of the mammalian target of rapamycin (mTOR) signalling pathway in both skeletal muscle and PDAC. Here, we test in the same murine PDAC model, in combination with gene expression microarray and gene set enrichment analysis, the hypothesis that β‐hydroxy‐β‐methylbutyrate (HMB), a metabolite of leucine, will stimulate mTOR activity and protein synthesis in skeletal muscle but will suppress (in lean or obese mice and either alone or in combination with gemcitabine chemotherapy) PDAC growth and/or tumour mTOR activity.

**Methods:** Male C57BL/6 mice received either control diet (*n* = 60) or a diet‐induced obesity (DIO) regimen (*n* = 60) for 10 weeks and then were subcutaneously injected (right flank) with Panc02 PDAC cells and further randomized to continue their diets ±HMB supplementation and ±gemcitabine treatment.

**Results:** We found that (i) DIO, relative to control diet, significantly increases Panc02 tumour growth (nearly 4‐fold); (ii) HMB significantly decreases tumour growth in DIO–fed mice but has no effect in control mice; (iii) cell proliferation (Ki‐67 positivity) was increased in tumours from DIO mice and reversed by HMB; (iv) HMB partially rescued gemcitabine responsiveness in DIO mice; and (v) HMB partially reverts the immune‐suppressive tumour microenvironment induced by obesity, particularly by increasing tumour‐infiltrating CD8+ T cell populations.

**Conclusions:** These preclinical findings suggest that HMB may have anti‐cachexia and anti‐tumour activity against PDAC, particularly in the context of obesity.

**2-14**

**MuRF2 as a novel regulator of muscle mass loss in cardiac cachexia**

**T. Scott Bowen** ^1^, Thanh Nguyen^2^, Sarah Werner^2^, Siegfried Labeit^3^ and Volker Adams^4^

^1^ *University of Leeds, Leeds, UK;* ^2^ *Leipzig Heart Center, Leipzig, Germany;* ^3^ *Universitätsklinikum Mannheim, Mannheim, Germany;* ^4^ *Dresden Heart Center, Dresden, Germany*

**Introduction:** The muscle‐specific ubiquitin E3 ligase muscle RING finger 1 (MuRF1) is a known mediator of muscle loss in numerous wasting conditions, which include heart failure, denervation, and critical illness. In contrast, much remains unknown about the second MuRF family member, MuRF2, and whether it plays any direct role in the regulation of muscle mass loss. The current study, therefore, investigated if genetic deletion of MuRF2 could attenuate muscle atrophy in a mouse model of cardiac cachexia.

**Methods:** Four groups of mice were compared: saline‐treated C57BL/6 wild‐type (WT) (*n* = 12) and global MuRF2 knockouts (KO) (*n* = 11) and monocrotaline‐treated WT (*n* = 9) and MuRF2 KO (*n* = 9) to induce cardiac cachexia. After 8 weeks of intervention, *in vitro* soleus muscle function and fibre cross‐sectional area was measured, while protein expression was assessed using western blot.

**Results:** Monocrotaline treatment induced cardiac failure in both WT and MuRF2 KO mice, with evidence of weight loss, pulmonary congestion, and right‐ventricular hypertrophy (*P* < 0.05). Muscle protein expression of MuRF2 was 72% higher (*P* < 0.05) in WT mice with cardiac cachexia compared to controls, which was associated with a reduction (*P* < 0.05) in soleus function by 16 ± 2%, wet‐weight by 11 ± 3%, and cross‐sectional area of the tibialis anterior by 32 ± 6%. In contrast, soleus function, wet‐weight, or tibialis anterior fibre cross‐sectional area remained unchanged (*P* < 0.05) when MuRF2 KO mice with cardiac cachexia were compared to control KO mice.

**Conclusions:** Genetic deletion of MuRF2 prevented muscle wasting in mice with cardiac cachexia induced by monocrotaline treatment. These data provide evidence, therefore, that MuRF2 can play a key role in the regulation of muscle mass loss induced by cardiac cachexia.

**2-26**

**MicroRNA‐regulated networks in skeletal muscle cells treated with interleukin‐6**

**Paula Paccielli Freire**, Geysson Javier Fernandez Garcia, Sarah Santiloni Cury, Grasieli de Oliveira, Letícia Oliveira Lopes, Leonardo Nazario de Moraes, Maeli Dal‐Pai‐Silva and Robson Francisco Carvalho

*Institute of Biosciences, São Paulo State University (UNESP), Botucatu, SP, Brazil*

Cachexia is a metabolic syndrome in patients with advanced cancer characterized by a progressive loss of skeletal muscle, resulting in weakness, diminished quality of life, and poor response to radio‐ and chemotherapy. Systemic inflammation contributes to the development of cancer‐cachexia (CC), and interleukin‐6 (IL6) has been implicated as a key pro‐inflammatory cytokine in the pathogenesis of muscle wasting in CC. Although global gene expression alterations in response to cytokines are informative, to our knowledge, no other study has examined the role of microRNAs (miRNAs) in skeletal muscle cells in response to IL6. Here, we described an integrative analysis between miRNA and mRNA expression profiles of C2C12 cells treated with IL6. We found that IL6 triggers atrophy, decreases the myoblasts fusion index, and reduces Myod protein levels in C2C12 myotubes. In addition, we identified 20 deregulated miRNAs in C2C12 myotubes in response to IL6, including the miR‐23a, miR‐146a, miR‐148b, and miR‐497. Gene ontology analysis on integrative mRNA/miRNA expression profiling data revealed miRNA interactions affecting genes that regulate cell differentiation, apoptosis, migration, and regulation of catabolic process. Interestingly, we also found that the miR‐497 potentially repress the expression of Id3, whose inhibitory effect on myogenic differentiation is well established. Thus, our integrative microRNA and mRNA analysis results identified microRNA‐regulated network and suggests a new regulatory axis of IL6/miR‐497/Id3 during the muscle atrophy in response to IL6 treatment.

Grant #12/13961‐6, São Paulo Research Foundation (FAPESP).

**2-27**

**Identification of potential cancer cachexia biomarkers by tumour transcriptome analysis in non‐small cell lung cancer patients with low muscularity**

**Sarah Santiloni Cury** ^1^, Diogo de Moraes^1^, Paula Paccielli Freire^1^, Douglas Marques Venâncio Pereira Marques^1^, Grasieli de Oliveira^1^, Geysson Javier Fernandez García^1^, Maeli Dal‐Pai‐Silva^1^, Érica Nishida Hasimoto^2^, Patrícia Pintor dos Reis^2^ and Robson Francisco Carvalho^1^

^1^ *Institute of Biosciences, São Paulo State University (UNESP), Botucatu, São Paulo, Brazil;* ^2^ *Department of Surgery and Orthopedics, School of Medicine, São Paulo State University (UNESP), Botucatu, São Paulo, Brazil*

**Introduction:** Cancer cachexia (CC) is a multifactorial syndrome characterized by an ongoing loss of skeletal muscle mass that leads to increased morbidity and poor prognosis. Up to 60% of all patients with non‐small cell lung cancer (NSCLC) develop significant loss of muscle mass as demonstrated by computed tomography (CT) analysis.

**Methods:** Here, we reanalysed CT images available on The Cancer Imaging Archive database and used transcriptome analysis aiming to identify new potential secreted molecules by the tumours of patients with low muscularity. Pectoralis muscle area from CT of 89 NSCLC patients (training set) was analysed to identify which ones had high or low muscularity.

**Results:** Using tumour microarray data from our training set, we identified 105 deregulated transcripts in patients with low muscularity. Among the 75 up‐regulated transcripts, we found several pro‐inflammatory cytokines, including *IL‐6*, *IL‐8*, and *CSF3*. Functional enrichment analysis using Gene Ontology Consortium demonstrated that up‐regulated genes were related to ‘cytokine activity’ and ‘extracellular matrix region’. In addition, computational analyses using SignalP, SecretomeP, and TargetP, Vesiclepedia and Human Cancer Secretome databases allowed us to identify up‐regulated transcripts *NCAM1*, *CNTN1*, *SCG2*, *CADM1*, *IL8*, *NPTX1*, and *APOD* as novel potential cachexia biomarkers in NSCLC secretome. Moreover, to verify the biomarkers prognostic value, we used tumour gene expression data to predict survival using seven additional NSCLC validation sets available in SurvExpress database. Noteworthy, this analysis demonstrated that our new potential biomarkers were capable to distinguish NSCLC patients with poor prognosis, and specifically, *IL8* was significantly associated with poor prognosis in all validation sets.

**Conclusions:** Integrative analysis of muscularity CT‐based data and transcriptome profiles identified cancer patients with low muscularity, from which the tumours expressed a set of cachexia‐related transcripts capable of predicting poor prognosis. These findings also reveal *IL8* as a potential prognostic biomarker in NSCLC patients associated with lower muscularity.

**Acknowledgments:** The results shown here are in part based upon data generated by the TCGA Research Network: http://cancergenome.nih.gov/. The following grant helped support the development of this work: São Paulo Research Foundation – FAPESP (grant *#17/21223‐9*).

**2-28**

**IL‐6 trans‐signalling among tumour, muscle, and fat mediates pancreatic cancer cachexia**

**Joseph Rupert** ^1^, Andrea Bonetto^1,4,5^, Ashok Narasimhan^2^, Leonidas Koniaris^2,4,5^ and Teresa Zimmers^1,2,3,4,5,6^

^1^ *Departments of Biochemistry and Molecular Biology, Indiana University School of Medicine, Indianapolis, IN, 46202, USA;* ^2^ *Departments of Surgery, Indiana University School of Medicine, Indianapolis, IN, 46202, USA;* ^3^ *Anatomy and Cell Biology, Indiana University School of Medicine, Indianapolis, IN, 46202, USA;* ^4^ *Otolaryngology Head and Neck Surgery, Indiana University School of Medicine, Indianapolis, IN, 46202, USA;* ^5^ *IUPUI Center for Cachexia Research, Innovation & Therapy (CCRIT);* ^6^ *IU Simon Cancer Center, Indianapolis, IN, 46202, USA*

Cachexia, the involuntary loss of fat, muscle and bone is associated with pancreatic ductal adenocarcinoma (PDAC) and contributes to the 5‐year mortality of >91%. Interleukin‐6 (IL‐6) is increased in the blood of patients with PDAC and correlates with increased cachexia and reduced survival. IL‐6 has been well documented, yet IL‐6 signalling mechanisms between tissues remain elusive. IL‐6 activates signal transduction by binding with the membrane bound IL‐6 receptor (IL‐6R) (classical signalling) or the soluble IL‐6R (sIL‐6R; trans‐signalling), produced from shedding of the membrane receptor. Here, we investigate the mechanisms of IL‐6 signalling between tumour, fat, and muscle and the subsequent effects on cachexia using isolated tumour cells from the LSL‐**K**rasG12D:LSL‐Tr**p**53R172H:Pdx1‐**C**re (KPC) genetic mouse model of PDAC. We used CRISPR/Cas9 editing to delete IL‐6 expression in KPC tumour cells (KPC IL‐6^KO^) and orthotopically injected mice with KPC or KPC IL‐6^KO^ cells.

KPC tumours expressed IL‐6 in tumour and stromal cells, while also causing increased plasma IL‐6 and sIL‐6R protein levels in mice. KPC tumour mice had increased IL‐6 gene and protein expression in adipose tissue but not muscle. Interestingly, while KPC tumour mice had increased IL‐6R gene but not protein expression in muscle, adipose tissue IL‐6R gene expression was unchanged while concomitant accumulation of sIL‐6R protein (55 kDa) was measured. Furthermore, increased plasma glycerol and fatty acids, augmented fat loss, reduced muscle mass and increased myosteatosis were observed in KPC tumour mice. Increased cachexia severity was largely due to tumour‐derived IL‐6, since KPC IL‐6^KO^ tumour mice had attenuated cachexia and increased survival without changes in tumour size or the use of an anti‐tumour therapy. Thus, our data implicate a feed‐forward signalling loop in PDAC cachexia, sparked by tumour‐derived IL‐6, resulting in skeletal muscle sIL‐6R production, which augments adipose tissue lipolysis via IL‐6 trans‐signalling and promotes lipotoxicity‐induced skeletal muscle wasting in PDAC.

**2-29**

**Deficiency in the immune inhibitory receptor LAIR‐1 enhances body weight loss and airway inflammation in an animal model for COPD.**

Kuldeep Kumawat^†, 1,2^, Ruben Geerdink^1^, Marije Hennus^3^, **Gert Folkerts** ^4^, Anita van Oort‐Jansen^1^, Alexandra Mazharian^5^, Steve P. Watson^5^, Frank Coenjaarts^6^, Louis Bont^1,7^ and Linde Meyaard^1,2^

^1^ *Laboratory for Translational Immunology, Department of Immunology, University Medical Center Utrecht, Utrecht, The Netherlands;* ^2^ *Oncode Institute, University Medical Center Utrecht, Utrecht, The Netherlands;* ^3^ *Department of Pediatric Intensive Care, Wilhelmina Children's Hospital, University Medical Center Utrecht, Utrecht, The Netherlands;* ^4^ *Division of Pharmacology, Faculty of Science, Utrecht Institute for Pharmaceutical Sciences, Utrecht University, Utrecht, The Netherlands;* ^5^ *Centre for Cardiovascular Sciences, Institute for Biomedical Research, College of Medical and Dental Sciences, University of Birmingham, Birmingham, UK;* ^6^ *Department of Medical Microbiology, University Medical Center Utrecht, Utrecht, The Netherlands;* ^7^ *Department of Pediatrics, Wilhelmina Children's Hospital, University Medical Center Utrecht, Utrecht, The Netherlands*

^†^Equal contribution

**Introduction:** Cachexia, muscle wasting, and inflammation are well recognized as features of chronic obstructive pulmonary disease (COPD), adversely affecting disease progression and prognosis. Importantly, muscle wasting not only contributes to diminished skeletal muscle function, reduced exercise capacity, and decreased health status but is also a determinant of mortality in COPD. Inflammation may be one of the triggers for body weight loss. Therefore, factors that modulate the chemotaxis and activation of immune cells could be key in both the loss of body weight and inflammation. We recently identified the collagen receptor LAIR‐1 as a functional inhibitory receptor on airway‐infiltrated neutrophils in viral bronchiolitis patients.^4^ We hypothesize that LAIR‐1 regulates neutrophilic driven airway diseases and hence body weight loss.

**Method:** LAIR‐1‐deficient (*Lair1* ^‐/‐^) or wild‐type mice were exposed to cigarette smoke for 10 days or infected with RSV as commonly accepted models of neutrophil‐driven lung inflammation. Mice were monitored for cellular airway influx, weight loss, and cytokine concentrations.

**Results:** LAIR‐1 deficiency significantly enhanced the cigarette smoke‐induced body weight loss (from Days 4 to 10) and neutrophil/macrophage influx into the airways (on Day 10) compared to wild types. After RSV infection, *Lair1* ^‐/‐^ mice also showed an enhanced neutrophil recruitment without affecting viral‐, CXCL‐1 (KC) and IL‐6 levels in the airways. LAIR‐1 Fc administration in wild type mice, which blocks ligand induced LAIR‐1 activation, recapitulated *Lair1* ^‐/‐^ observations.

**Conclusions:** The immune inhibitory receptor LAIR‐1 limits the loss in body weight and neutrophil‐driven airway inflammation. Following our recent observations in humans^4^, we conclude that LAIR‐1 is a promising target for pharmacological intervention in such pathologies.

Reference4

Geerdink
RJ
, 
Hennus
MP
, 
Westerlaken
GHA
, 
Abrahams
AC
, 
Albers
KI
, 
Walk
J
, et al. LAIR‐1 limits neutrophil extracellular trap formation in viral bronchiolitis. J Allergy Clin Immunol
2018;141(2):811–4.2905097210.1016/j.jaci.2017.08.031

**2-30**

**Characterization of the endotoxin induced cachexia preclinical model**

Srinath Jagarlapudi, Stephanie Joaquim, Autumn Arons, Xiangping Li, Yao Zhang, Christian Cortes, Bei Betty Zhang, Zhidan Wu, Jean‐Philippe Fortin and **Olivier Bezy**

*Internal Medicine Research Unit, Pfizer, 1 Portland Street, Cambridge, MA, 02139, USA*

**Introduction:** Cancer cachexia is a complex metabolic syndrome associated with loss of muscle with or without loss of fat mass. Anorexia, inflammation, and increased muscle protein breakdown are frequently associated with cachexia.

**Method:** Endotoxin mediated sepsis has been suggested to recreate in an acute way most of the features of cancer cachexia. We decided to test this model in order to establish the best formulation and dose of bacterial lipopolysaccharides (LPS) adequate to obtain reproducible cachexia phenotypes in preclinical models.

**Results:** Our data suggest that dilution of LPS in water followed by resuspension in saline solution containing bovine serum albumin produced the most reproducible cachexia based on cytokines induction, food intake, body weight, and skeletal muscle loss. Furthermore, at 250mpk, LPS was able to produce a reduction in lean mass which persisted 72 h post LPS administration. Interestingly, reduction of food intake caused by LPS treatment was not responsible for the loss of lean mass as demonstrated in pair‐feeding experiments. Correlation analysis between plasma cytokines 3 h post LPS dose and cachexia features demonstrated strong associations with doses of LPS administered and the extent of body weight and muscle mass loss. Finally, we report that several cytokines, including TNF, IL‐6, KC/GRO, and GDF15, were acutely induced by LPS treatment and quantitatively correlated with the severity of weight loss.

**Conclusions:** Endotoxins induced cachexia is a potent and reproducible preclinical model capturing many aspects of cancer cachexia such as anorexia, loss of body weight, and lean mass as well as inflammation.

**2-31**

**Impact of ERK silencing on muscle cell culture proliferation**

**Aline R.R. Lima** ^1^, Luis H. Zucoloto^1^, Paula P. Freire^2^, Mariana J. Gomes^1^, Luana U. Pagan^1^, Sarah Cury^2^, Éder A. Rodrigues^1^, Robson F. Carvalho^2^, Katashi Okoshi^1^ and Marina P. Okoshi^1^

^1^ *Botucatu Medical School, São Paulo State University – UNESP, São Paulo, Brazil;* ^2^ *Botucatu Bioscience Institute, São Paulo State University – UNESP, São Paulo, Brazil*

**Introduction:** The mitogen‐activated protein kinase family, especially ERK, is necessary for skeletal muscle mass and phenotype maintenance. Although rats with diseases such as heart failure and cancer present muscle atrophy and reduced ERK expression, the role of ERK in these processes is poorly understood. In previous study, we observed that ERK silencing in C2C12 cells decreases myoblasts number, declines myoblast differentiation into myotube index, and reduces myofibers area and diameter. Purpose: The study aims to identify the impact of a decrease in skeletal muscle ERK protein in C2C12 muscle cell culture proliferation. Methods: ERK gene silencing was performed by specific RNA interference (siRNA‐ERK). Myoblasts proliferation was analysed by MTT (Thiazolyl Blue Tetrazolium Blue) assay. Proliferation test was performed at 0, 12, 24, 36, and 48 h after gene silencing. Cell proliferation index was estimated by absorbance variation in which moment and initial absorbance in relation to time variation. Comparison between groups was performed by Student's *t* test. Results: Data are shown in the table. We did not observe statistical difference between groups in any analysed variables. Conclusion: Although ERK has an essential role in myoblast differentiation into myotube, ERK reduction does not impact cellular proliferation in C2C12 cells.

**Table:** 

	Control	siRNA‐ERK
0 h	0.247 ± 0.05	0.184 ± 0.02
12 h	0.306 ± 0.04	0.232 ± 0.03
24 h	0.449 ± 0.07	0.348 ± 0.05
36 h	0.489 ± 0.09	0.425 ± 0.05
48 h	0.505 ± 0.06	0.441 ± 0.04
Δ/time	1.00 ± 0.25	0.90 ± 0.21

*Control*: normal C2C12 cells, n = 3; siRNA‐ERK: ERK gene silencing C2C12 cells, n = 3; Δ/time: proliferation index in relation to time; data are expressed in absorbance units; mean ± standard deviation, *P* > 0.05.

**3-01**

**The expression landscape of cachectic factors across different tumour types predicts cancer outcomes**

Paula Paccielli Freire^1^, Geysson Javier Fernandez Garcia^1^, Sarah Santiloni Cury^1^, Diogo de Moraes^1^, Maeli Dal Pai‐Silva^1^, Silvia Regina Rogatto^2^ and **Robson Francisco Carvalho** ^1^

^1^ *Institute of Biosciences, São Paulo State University (UNESP), Botucatu, Brazil;* ^2^ *Department of Clinical Genetics, Vejle Hospital and Institute of Regional Health Research, University of Southern Denmark, Vejle, Denmark*

**Introduction:** Cancer cachexia is a multifactorial syndrome characterized by muscle wasting, leading to a significant weight loss that impacts patient morbidity and survival. Cachexia is highly associated with specific tumour types, such as pancreatic, esophageal, gastric, lung and liver, and the causes of variation in cachexia prevalence and severity are still unknown. While distinct circulating mediators (soluble cachectic factors) derived from tumour cells have been implicated with the pathogenesis of the syndrome, these associations were generally based on plasma concentration rather than tumour tissue‐specific gene expression levels. The access to The Cancer Genome Atlas (TCGA) database has allowed the identification of gene expression profiles that correlate with survival following cancer prognosis in different tumour types. Thus, we hypothesized that tumour gene expression profiling of cachectic factors could reveal potential cancer‐specific biomarkers of clinical outcome.

**Methods:** Here, we systematically investigated the tumour expression landscape of cachectic factors as potential predictive markers of overall survival in 12 tumour types from TCGA. We also compared the expression profile of cachectic factors between tumour and normal tissues (4651 and 2737, respectively) using uniformly processed RNA sequencing data from Genotype‐Tissue Expression (GTEx) and TCGA databases.

**Results:** Interestingly, cachectic factors presented a tumour‐specific expression profile. Furthermore, a distinct gene signature according to tumour type was significantly associated with poor prognosis. The number of up‐regulated cachectic factor genes in tumour compared to normal tissues was strongly correlated with the prevalence of cachexia and weight loss (average percentage) across tumour types; and this finding could be useful to explain why specific cancer types are more likely to develop cachexia.

**Conclusions:** Our results demonstrate that the expression profile of cachectic factors predicts cancer outcomes. A tumour‐specific gene expression profile of cachectic factors has potential to contribute to early diagnosis, prognosis and treatment of cancer cachexia.

**Acknowledgements:** The results shown here are in whole based upon data generated by the TCGA Research Network: http://cancergenome.nih.gov/, and by the GTEx Portal (https://gtexportal.org/). The following grants helped support the development of this work: São Paulo Research Foundation – FAPESP (grants 12/13961‐6 and 13/50343‐1).

**3-02**

**Targeting interleukin‐1α (IL‐1α) in cancer cachexia: a narrative review**

**James McDonald** ^1^ and Barry Laird^2^

^1^ *Edinburgh Medical School, The University of Edinburgh, Edinburgh, United Kingdom;* ^2^ *Institute of Genetics and Molecular Medicine, The University of Edinburgh, Edinburgh, United Kingdom*

Cachexia is defined as ongoing loss of skeletal muscle mass, with or without depletion of adipose tissue. It is a common syndrome in cancer patients and affects 50% of those who are diagnosed. Cachexia, which cannot be fully reversed and causes significant functional impairment is caused by various mechanisms such as an altered energy balance and disruption of homeostatic control by the CNS. The systemic inflammatory response (SIR) is central to cachexia development and recent guidelines from ESPEN have advocated using markers of the SIR in cachexia assessment.

A multitude of pro‐inflammatory cytokines are involved in the SIR in cachexia. Of these, IL‐1α has been identified as a key cytokine, and its role as a therapy has been explored. This review will explore the role of IL‐1α in the genesis and maintenance of cachexia, and its potential as a therapeutic target. A summary of clinical trials which have examined antibodies to IL‐1α will also be presented.

**3-08**

**Sarcopenia or frailty as predictors of chemotherapy toxicity in elderly cancer patients? The ONCOSARCO project**

**Maria‐José Molina‐Garrido** ^1^, Maria‐Carmen Soriano‐Rodríguez^2^, Carmen Guillén‐Ponce^3,4^ and Borja‐Manuel Fernández‐Félix^5,6^

^1^ *Consulta de Cáncer en el Anciano, Sección de Oncología Médica, Hospital General Virgen de la Luz, Cuenca, Spain;* ^2^ *Sección de Oncología Médica, Hospital General Virgen de la Luz, Cuenca, Spain;* ^3^ *Servicio de Oncología Médica, Hospital General Universitario Ramón y Cajal, Madrid, Spain;* ^4^ *Instituto de Investigación IRYCIS;* ^5^ *Unidad de Bioestadística Clínica, Hospital General Universitario Ramón y Cajal, Madrid, Spain;* ^6^ *Instituto de Investigación IRYCIS*

**Introduction:** The ONCOSARCO Project is attempting to (i) find out which variable (frailty or sarcopenia) is the better predictor of chemotherapy toxicity in the elderly cancer patient, and (ii) identify new predictive parameters of toxicity by using variables related to muscle mass, muscle strength, and physical function.

**Material and Methods:** Before the start of chemotherapy treatment, their muscle mass (skeletal muscle mass index), muscle strength (handgrip, spherical grip and pinch gauge, knee extension strength, and hip flexion), and physical function (gait speed and five sit‐to‐chair test) were all registered in older patients (≥70 years) with cancer. The criteria used were those of the *European Working Group on Sarcopenia in Older People* (EWGSOP) to define sarcopenia and the phenotype of frailty, as described by Linda Fried, to detect frailty. The final event was considered the appearance of severe toxicity; and, as competitive event, the absence of severe toxicity but in patients who had not completed 4 months of chemotherapy, using a multinomial logistical regression analysis.

**Results:** A total of 103 patients have severe toxicity in 58.3%. In the multinomial logistical regression analysis, frail patients were at greater risk of developing toxicity than pre‐frail patients (RRR: 9.3; 95% CI: 2.1–41.2; *P* = 0.003) and so male patients were (RRR: 17.2; 95% CI: 1.9–152.4; *P* = 0.011). Sarcopenia was not a good indicator of toxicity. A predictive model of toxicity was sought using the variables of muscle mass, muscle strength, and physical function. The only variable associated significantly with severe toxicity was knee extension strength (RRR 0.7; 95% CI: 0.5–0.9; *P* = 0.031).

**Conclusions:** In the field of Geriatric Oncology frail patients, but not sarcopenic patients, are at 9.3 times more risk of developing toxicity than pre‐frail patients. Knee extension strength is associated in a significant way with chemotherapy toxicity. Future studies should be able to confirm its role as a predictor of toxicity in the elderly cancer patient.

**3-27**

**Differential structural features in soleus and gastrocnemius of carnitine treated cancer cachectic rats**

Anna Salazar‐Degracia^1^, **Sílvia Busquets** ^3,4^, Roberto Serpe^5^, Josep M. Argilés^3,4^, Francisco J. López‐Soriano^3,4^ and Esther Barreiro^1,2^

^1^ *Pulmonology Department‐Muscle Wasting and Cachexia in Chronic Respiratory Diseases and Lung Cancer Research Group, IMIM‐Hospital del Mar, Parc de Salut Mar, Health and Experimental Sciences Department (CEXS), Universitat Pompeu Fabra (UPF), Barcelona Biomedical Research Park (PRBB), Barcelona, Spain;* ^2^ *Centro de Investigación en Red de Enfermedades Respiratorias (CIBERES), Instituto de Salud Carlos III (ISCIII), Barcelona, Spain;* ^3^ *Cancer Research Group, Departament de Bioquímica i Biomedicina Molecular, Facultat de Biologia, Universitat de Barcelona, Barcelona, Spain;* ^4^ *Institut de Biomedicina de la Universitat de Barcelona (IBUB), Barcelona, Spain;* ^5^ *Department of Medical Sciences and Public Health “M. Aresu”, University of Cagliari, Cagliari, Italy*

**Introduction:** Muscle mass loss and wasting are associated with chronic respiratory and cardiac conditions and cancer.

**Methods:** We hypothesized that L‐carnitine treatment may differentially revert muscle fibre atrophy and other structural alterations (muscle damage) in slow‐ and fast‐twitch limb muscles of rats bearing the Yoshida ascites hepatoma. In soleus and gastrocnemius of tumour‐bearing rats (10^8^ AH‐130 Yoshida ascites hepatoma cells inoculated intraperitoneally) with and without treatment with L‐carnitine (1 g/kg body weight for 7 days, intragastric), fibre typing and morphometry, damage, proteolytic, and signalling markers were explored.

**Results:** Carnitine treatment improved atrophy of slow‐ and fast‐twitch fibres (gastrocnemius particularly), structural abnormalities (both muscles), and attenuated proteolytic and signalling markers (gastrocnemius). Carnitine improved muscle atrophy and proteolysis in a muscle‐specific manner in cancer‐induced cachexia.

**Conclusions:** These data reveal the need to study muscles of different fibre type composition and function to better understand whereby carnitine exerts its beneficial effects on skeletal muscles in muscle wasting processes.

**3-28**

**miRNA transcriptome is altered in the skeletal muscle and in plasma‐derived microvesicles during experimental cancer cachexia**

**Marc Beltrà** ^1,2^, Fabrizio Pin^1,2^, Serena De Lucia^1,2^, Riccardo Ballarò^1,2^, Giovanni Birolo^3,4^, Barbara Pardini^3,4^, Giuseppe Matullo^3,4^, Fabio Penna^1,2^ and Paola Costelli^1,2^

^1^ *Department of Clinical and Biological Sciences, Experimental Medicine and Clinical Pathology Unit, University of Turin, Turin, Italy;* ^2^ *Interuniversity Institute of Myology (IIM), Urbino, Italy;* ^3^ *Italian Institute for Genomic Medicine, IIGM (formerly Human Genetics Foundation, HuGeF), Turin, Italy;* ^4^ *Department of Medical Sciences, University of Turin, Turin, Italy*

MicroRNAs (miRNAs) exert essential functions in many tissues, playing a central role in proper muscle cell commitment, proliferation, and differentiation. Moreover, some of those short RNA sequences can be secreted in blood clotted in extracellular vesicles (microvesicles and exosomes) and transported to distant tissues, where they can convey molecules endowed with signalling functions.^5^ Interestingly, the induction of catabolic conditions in skeletal muscle is correlated with miRNAome alterations.^6^ Besides muscle, circulating miRNAs can also be modulated in certain pathologies, therefore being good potential candidates for diagnosis and treatment of muscle diseases.

In the attempt to characterize the miRNA pattern in a cancer cachexia setting, we isolated total RNA from *gastrocnemius* muscle and plasma‐derived microvesicles of both healthy and cachectic mice bearing the transplantable C26 colon carcinoma. As for plasma, samples of two mice were pooled and consecutive centrifugations were applied for microvesicle isolation. Next‐generation sequencing (Illumina) was used to sequence whole miRNA transcriptome. A total of 304 miRNAs in skeletal muscle and 118 in plasma‐derived microvesicles were detected. About 30 miRNAs were differentially regulated in the skeletal muscle of tumour‐bearing mice, including the muscle‐specific miR‐133a. By contrast, miRNAs contained in plasma‐derived microvesicles appear poorly modulated in the C26 hosts. These results present new insight in the modulation of muscle and circulating miRNA expression during cancer cachexia.

References5

He
WA
 et al., Proc Natl Acad Sci U S A., 2014.6

Soares
RJ
 et al., J Biol Chem., 2014
10.1074/jbc.M114.561845PMC413920924891504

**3-29**

**Myeloid‐derived suppressor cells accumulation correlates with cancer cachexia development**

**Lingbing Zhang** and Geng Liu

*Yinuoke, Davis, CA, USA*

**Introduction:** Despite intensive efforts in the last decade to understand mechanisms underlying each individual symptom of cancer cachexia, therapeutic products targeting those mechanisms have failed in clinical trials suggesting the fundamental mechanism(s) causing cancer cachexia has not been identified. Although the role of myeloid‐derived suppressor cells (MDSC) has never been studied in cancer cachexia, elevated neutrophil/lymphocyte ratio (NLR) has been recognized as a reliable prognostic marker for outcomes of advanced cancer patients who usually have cachexia. In this study, we investigated the role of MDSC during cancer cachexia development.

**Methods:** Seven‐week old BALB/c mice were inoculated subcutaneously with 1 × 10^6^ C26 colon adenocarcinoma cells. Mice body weight were measured after tumour inoculation. Percentage of MDSC and lymphocytes in peripheral blood was monitored with flow cytometry after tumour inoculation. MDSC infiltration in organs was examined with flow cytometry and immunohistochemistry at different time points during cachexia progress.

**Results:** Compared with naive mice, mice with cachexia (5–10% body weight loss) have significantly elevated percentage of MDSC (Gr1^+^&CD11b^+^) in peripheral blood (7.90 ± 0.727 vs 72.15 ± 4.143, *P* < 0.0001). MDSC percentages in mice with severe cachexia (>15% body weight loss) further increase compared to mice with early stage cancer cachexia (72.15 ± 4.143 vs. 93.83 ± 1.971, *P* = 0.0032). In line with the dramatic percentage increase in peripheral blood, liver, lung, and spleen of mice under severe cachexia show massive MDSC infiltration by immunohistochemistry and flow cytometry examination (normal liver MDSC%: 1.533 ± 0.285 vs. cachectic liver MDSC%: 24.48 ± 2.782, *P* = 0.0008). More importantly therapeutic interventions targeting MDSC ameliorate cachexia symptoms.

**Conclusions:** Severe immune disorder characterized by accumulation of MDSC in peripheral blood and vital organs correlates with cancer cachexia development. Thus, targeting the excessive MDSC might represent a fundamental approach to the treatment of cancer cachexia.

**3-30**

**An innovative experimental set‐up for characterizing *in vivo* muscle weakness in mouse models of cancer cachexia**

Peggy Del Carmine, Bénédicte Chazaud and **Julien Gondin**

*Institut NeuroMyoGène (INMG), CNRS 5310 – INSERM U1217 ‐ UCBL1, Lyon, France*

**Introduction:** Cancer cachexia is a devastating syndrome characterized by severe skeletal muscle wasting (i.e. atrophy) that leads to muscle weakness (i.e. reduced force production). Several therapeutic strategies have been developed to counteract the deleterious consequences of cachexia. A reliable evaluation of muscle force is therefore required to carefully assess their effectiveness. So far, preclinical studies typically used either invasive (e.g. attachment of distal tendon to a transducer) or non‐specific (e.g. grip strength) approaches to record force production. Here, we describe a non‐invasive experimental device allowing reproducible and longitudinal force measurements in plantar flexor mouse muscles.

**Methods:** The strictly non‐invasive experimental set‐up (NIMPHEA_Research, AII Biomedical SAS, Grenoble, France) incorporates four distinct components allowing force measurements, non‐invasive muscle stimulation, prolonged anaesthesia, and animal thermoregulation. Plantar flexor muscles force measurements were achieved with the foot positioned on a pedal of the ergometer. Electrical stimuli were delivered through two surface electrodes located below the knee and the Achille's tendon. Maximal force production (F_max_) was recorded in response to a 100 Hz tetanic train. F_max_ was first recorded twice over a 2‐day period in control mice to investigate the reproducibility of measurements. Balb/C mice were then used and inoculated subcutaneously in the back with either 5 × 10^5^ C26 cells or PBS. F_max_ was recorded before and from 7 to 14 days after injection. Plantar flexor muscles were harvested and weighed at Day 14.

**Results:** We found a high reproducibility of F_max_ measurements (CV = 5%). F_max_ gradually decreased in C26 mice and reached 62% of baseline values at Day 14, illustrating the occurrence of severe muscle weakness. Changes in F_max_ were positively correlated with plantar flexor muscles mass.

**Conclusions:** This non‐invasive experimental set‐up represents a major advance for monitoring the progression of muscle weakness but also for assessing the efficacy of therapeutic interventions in mouse models of cancer.

**3-32**

**Exercise capacity in patients with advanced cancer: the role of cardiovascular function**

**Christine Diehl** ^1^, Ruben Evertz^1^, Breno Godoy^1^, Annalen Bleckmann^2^, Ute König^3^ and Stephan von Haehling^1^

^1^ *Department of Cardiology and Pneumology, University Medical Center Göttingen, Göttingen, Germany;* ^2^ *Department of Hematology and Medical Oncology, University Medical Center Göttingen, Göttingen, Germany;* ^3^ *Department of Gastroenterology and Gastrointestinal Oncology, University Medical Center Göttingen, Göttingen, Germany*

**Introduction:** Patients with advanced cancer have been shown to display cardiovascular perturbations that appear independently of ongoing cardiotoxic chemotherapy. These perturbations may be involved in the development of clinical symptoms such as fatigue, shortness of breath, or impaired exercise capacity. We sought to determine influencing factors of exercise capacity in patients with advanced cancer.

**Methods:** A total of 102 patients with solid tumours [age 61 ± 11 years; 51% male; body mass index [BMI] 24.8 ± 4.6 kg/m^2^; cachexia 51.5%; New York Heart Association [NYHA] class ≥2: 54.9%; median Karnovsky Index 90%] were prospectively recruited at the University of Göttingen Medical Center from October 2017 until September 2018. All participants underwent 24‐h ECG to assess heart rate variability (HRV) using the standard deviation of the normal to normal interval (SDNN) value. Quality of life (QoL) was evaluated using the EQ‐5D questionnaire. Exercise capacity was assessed using the 6‐min walk test (6‐MWT) in 81 patients. Cachexia was defined as weight loss exceeding 5% of body weight over a period less than 12 months or a loss exceeding 2% of body weight when baseline BMI was 20 kg/m^2^ or less.

**Results:** The 6‐MWT distance ranged from 120 to 690 m with the median of the population being 464 m [interquartile range (IQR), 392–537] and the mean being 449 ± 128 m. SDNN ranged from 31 to 232 ms (median 105, mean 107). The 6‐MWT correlated with age (*R* = −0.46), BMI (*R* = −0.25), NYHA class (*R* = −0.57), Karnovsky Index (*R* = 0.65), systolic blood pressure (*R* = −0.42), SDNN index (*R* = 0.35), very low frequency (VLF) power (*R* = 0.37), Hb (*R* = 0.25), and QoL (*R* = 0.62) (all *P* < 0.05). Using multivariable linear regression, we found that the 6‐MWT distance correlated with NYHA class [standardized coefficient (SC) = −0.47, *P* < 0.001], systolic blood pressure (−0.28, *P* = 0.001), sympathetic activity (0.17, *P* = 0.04), and age (−0.21, *P* = 0.02), but not with BMI or SDNN. In a second model, we found that SDNN in combination with BMI, QoL, and systolic blood pressure can closely predict the outcome of 6‐MWT (*R* ^2^ = 0.54, *P* < 0.001).

**Conclusions:** Cardiovascular function parameters serve as independent predictors of exercise capacity as assessed using the 6‐MWT in patients with advanced cancer. Further studies with larger number of participants are required to better understand the regulation and intervention possibilities in these patients.

**4-16**

**Discovery and development of first‐in‐class USP19 inhibitors with *in vivo* muscle sparing activity**

**Xavier Jacq** ^1^, Gerald Gavory^1^, Colin O'Dowd^1^, Nathalie Bedard^2,3^, Oliver Barker^1^, Christina Bell^1^, Stephanie Burton^1^, Eamon Cassidy^1^, Joana Costa^1^, Ashling Henderson^1^, Matt Helm^1^, Peter Hewitt^1^, Caroline Hughes^1^, Mary McFarland^1^, Hugues Miel^1^, Lauren Proctor^1^, Shane Rountree^1^, Rachel Church^1^, Ewelina Rozycka^1^, Mark Wappett^1^, Steven Whitehead^1^, Simon S. Wing^2,3^ and Tim Harrison^1^

^1^ *Almac Discovery, Centre for Precision Therapeutics, 97 Lisburn Road, Belfast, BT9 7AE, UK;* ^2^ *Department of Medicine, McGill University and McGill University Health Centre, Montreal, Quebec, Canada;* ^3^ *Montreal Diabetes Research Centre, Montreal, Quebec, Canada*

Over the past decade, protein ubiquitination has emerged as an important post‐translational modification with regulatory functions in all important cellular processes. Deubiquitinating enzymes (DUBs) including ubiquitin specific proteases (USPs) are cysteine proteases that catalyse the de‐ubiquitination of protein substrates, hence regulating their levels and/or function.

As a result of their increasing implications in the aetiology of numerous pathological conditions including neurodegeneration, metabolic disorders, and cancer, DUBs represent an attractive and promising target class for the development of innovative medicines with high therapeutic impact. However, despite 15 years of intense research, DUBs have proved largely refractory to drug discovery efforts.

USP19 has recently emerged as a potentially important target in muscular atrophy and various other disorders involving aberrant protein quality control. Herein, we describe the application of our Ubi‐*Plex*™ drug discovery platform to the identification and optimization of DUB inhibitors. In particular, we will highlight the versatility and robustness of Ubi‐*Plex*™ by describing the outcome of our focussed library screening, hit identification, and optimization activities on USP19.

A series of novel and highly potent (e.g. IC_50_ < 5.0 nM) and reversible USP19 inhibitors have been identified. Further profiling has demonstrated excellent selectivity against a large panel of DUBs and other non‐related enzymes (e.g. kinases and proteases). These inhibitors are cell‐permeable and exhibit potent target engagement in muscle cells with EC_50_ values <30 nM. Finally, we will describe the development of lead molecules with drug‐like properties which have allowed us to establish proof‐of‐concept efficacy studies in a denervation model of muscle wasting *in vivo*.

In summary, this work further exemplifies the tractability of the DUB target family and reports the discovery and detailed profiling of first‐in‐class inhibitors of USP19. These molecules support the rationale to target USP19 for muscle wasting disorders.

**5-13**

**The FORCE trial: focus on reducing dose‐limiting toxicities in colon cancer with resistance exercise: baseline functional status**

**Bette Caan** ^1^, Jeffrey A. Meyerhardt^2^, Elizabeth M. Brighton^2^, Justin C. Brown^2^, Kristin L. Campbell^3^, Adrienne Castillo^1^, Elizabeth M. Cespedes Feliciano^1^, Valerie S. Lee^1^, Charles P. Quesenberry^1^, Sara K. Quinney^4^, Barbara Sternfeld^1^, Renate M. Winkels^5^ and Kathryn H. Schmitz^5^

^1^ *Kaiser Permanente of Northern California, Oakland, CA, USA;* ^2^ *Dana Farber Cancer Institute, Boston, MA, USA;* ^3^ *University of British Columbia, Vancouver, Canada;* ^4^ *Indiana University School of Medicine, Indianapolis, IN, USA;* ^5^ *Penn State College of Medicine, Penn State Cancer Institute, Hershey, PA, USA*

**Background:** Low muscle mass has been associated with increased chemotherapy toxicities and worse colon cancer‐specific and overall mortality. Maintaining dose intensity, with less dose delays and dose reductions, can improve survival outcomes in colon cancer patients. Our primary goal of this ‘trial in progress’ is to determine if strength training during adjuvant chemotherapy will increase average relative dose intensity of individual drugs and combined chemotherapy regimens.

**Study Design:** FORCE will randomize 180 newly diagnosed Stage II and III colon cancer patients receiving chemotherapy with a fluoropyrimidine [5‐fluorouracil (5‐FU) or capecitabine (CAP)] +/− oxaliplatin (OX) from Kaiser Permanente of Northern California (KPNC), the Penn State Cancer Institute (PSCI), and the Dana Farber Cancer Institute (DFCI) to either strength/resistance training (RT) or waitlist control. Patients will receive four to six in‐person training sessions over the course of chemotherapy and will complete two training sessions per week at home until the completion of chemotherapy. Data on body composition, dietary intake, quality of life, fatigue, and functional status will be collected at both baseline and post‐intervention. We will examine between group differences for RT vs. waitlist control for relative dose intensity. We will also study changes in muscle mass (MM) and changes in specific inflammatory markers (e.g. CRP, IL‐6, and TNF‐RII) as potential markers of change in response to RT.

**Trial Results to Date:** We randomized 31 (18%) out of the 174 patients who met initial eligibility criteria. In looking at the first 31 patients recruited, we report 61.3% are male, 90.3% are Stage III, and 54.9% are receiving FOLFOX. Mean (SD) age and BMI is 57.2 (15.1) years and 26.9 (6.2) kg/m^2^, respectively. At baseline, the median (interquartile range) gait speed is 1.0 (0.91–1.29) m/s; the repeated chair stand (five times) is 12.2 (9.7–14.0) s; and the dominant hand grip strength for females is 28.5 (26.5–32.8) kg, and for males is 42.0 (35.0–60.0). On the short physical performance battery, 25% of the patients scored <10, indicating one or more mobility limitations. We will further report on baseline body composition from DXA and from CT, and the relationship of body composition measures to functional status measures.

**Conclusions:** Newly diagnosed non‐metastatic colon cancer patients undergoing chemotherapy who volunteer to participate have adequate functional status to engage in a strength training intervention.

**6-13**

**Impact of protein intake and high‐fat diet on muscle protein synthesis and lipid infiltration in relation to aging in rats**

**Eleonora Poggiogalle** ^1,2^, Fanny Rossignon^1^, Aude Carayon^1^, Jérome Salles^1^, Christophe Giraudet^1^, Jean‐Paul Rigaudière^1^, Sarah de Saint‐Vincent^1^, Felipe Sanchez^1^, Olivier LeBacquer^1^, Frédéric Capel^1^, S. Walrand^1^, Y. Boiri^1^ and C. Guillet^1^

^1^ *Unité de Nutrition Humaine, UMR 1019 INRA/UCA, CHU service de Nutrition Clinique, Clermont‐Ferrand, France;* ^2^ *Department of Experimental Medicine, Food Science and Human Nutrition Research Unit, Sapienza University, Rome, Italy*

**Introduction:** Ectopic lipid deposition impairs muscle anabolic response especially during aging. We hypothesized that the anabolic efficiency of dietary protein in skeletal muscle might be affected by high‐fat diet. The objective of the study was to investigate muscle protein synthesis in response to two levels of protein intake combined to two levels of fat intake.

**Methods:** Two groups of 60 adult and 49 old male Wistar rats were randomly divided into four groups: isocaloric standard diet (12% protein, 14% lipid, as STD12); isocaloric standard (high‐protein) diet (25% protein, 14% lipid, STD25); hypercaloric high‐fat (normal‐protein) diet (12% protein, 45% lipid, HFD12); and hypercaloric high‐fat (high‐protein) diet (25% protein, 45% lipid, HFD25). The nutritional intervention lasted 10 weeks. The fractional synthesis (FSR) and absolute synthesis rates (ASR) of mixed muscle proteins were calculated using isotopically labelled ^13^C‐Valine incorporation in tibialis anterior (TA). Muscle lipid content was assessed using chromatography method.

**Results:** Rats in the high‐fat diet groups self‐limited their food intake, so that energy intake was not different among the groups. Regardless of dietary intervention, TA weight was lower in old groups compared to their adult counterparts (all *P* < 0.01). FSR was lower in old rats fed the HFD25 compared to the old STD12 group (diet effect: *P* = 0.02), whereas FSR in old groups was higher than adult groups (age effect, all *P* < 0.05). No differences emerged in ASR between groups. Only old rats in the HFD12 group exhibited increased intramuscular triacylglycerols (age effect: *P* = 0.02; diet effect: HFD12 vs. STD 12: 2.04 ± 1.74 vs. 0.83 ± 0.49 μg/g, *P* = 0.02).

**Conclusions:** Aging is characterized by reduced muscle weight despite increased FSR, suggesting specific alterations in the nutritional regulation of muscle protein turnover. In isocaloric conditions, higher protein intake modulates muscle lipid infiltration, without improving age‐related anabolic resistance in old rats fed a high‐fat diet.

**6-14**

**Prognostic impact of nutritional supplementation for pancreatic cancer patients with cachexia at the time of cancer diagnosis**

**Seiko Miura**, Nobuhiko Ueda and Takeo Kosaka

*Department of General and Digestive Surgery, Kanazawa Medical University, Uchinada, Japan*

**Introduction:** Pancreatic cancer patients are often found to have cachexia. We speculate that nutritional status may impact therapeutic response and clinical outcome.

**Objective:** The study aims to examine the relationship between the invasiveness of cancer treatment and presence of cachexia and to investigate the efficacy of nutritional supplementation in improving the long‐term prognosis.

**Methods:** Restrospectively cases were divided into four groups based on the presence of cachexia and whether they received nutritional supplementation. Nutritional supplementation includes supplements introduced via IV, feeding tubes, as well as fortified blood products, and in‐person instruction from certified nutritionists; 105 pancreatic cancer cases receive the following treatments: 20 pancreaticoduodenectomy (PD), 24 distal pancreatectomy (DP), 44 radiation therapy with S‐1(CRT), 87 chemotherapy, and 9 palliative care.

**Results:** (i) There are 29.5% of the pancreatic cancer patients were found to have cachexia (74 without cachexia, 36 with, and 38 without supplementation; 31 with cachexia and 13 with and 18 without supplementation); (ii) of the 74 cases without cachexia, the mean survival for those receiving nutritional supplementation was 1317 days, compared to the 432 days for the control group (Log rank 8.954; *P* = 0.003). Of the 31 cases with cachexia, there were no statistical significance; (iii) DP puts the hazard ratio at 0.16—this implies that they are 6.25 times more likely to survive. Likewise, if we perform PD, the hazard ratio becomes 0.246—this group is 4.06 times more likely to survive. Nutritional supplementation, which is *P* < 0.078, does not appear to be statistically significant, but we can appreciate that the hazard ratio is at 0.604—this group is 1.65 times more likely to survive.

**Conclusions:** Our data support that lack of cachexia at the time of diagnosis is a strong positive predictor of clinical outcome for pancreatic cancer patients. Alternative treatment strategies must be considered for those with cachexia at the time of diagnosis.

**6-16**

**Neck circumference as undernutrition indicator in elderly nursing home residents**

**Alejandro Sanz‐Paris** ^1,2^, Beatriz Lardies‐Sanchez^1^, Javier Perez‐Nogueras^3^, Antonio Serrano‐Oliver^4^, Elena Torres‐Anoro^5^, A. Sanz‐Arque^6^ and Jose M. Arbones‐Mainar^2,7,8^

^1^ *Nutrition Department, University Hospital Miguel Servet, Zaragoza, Spain;* ^2^ *Research Institute in Zaragoza, Instituto de Investigación Sanitaria Aragón (IIS‐Aragón), Zaragoza, Spain;* ^3^ *Geriatric Unit, Elias Martinez Nursing Home, Zaragoza, Spain;* ^4^ *Geriatric Unit, Casa Amparo Nursing Home, Zaragoza, Spain;* ^5^ *Geriatric Unit, Romareda Nursing Home, Zaragoza, Spain;* ^6^ *General Practitioner, Tudela Health Center, Tudela, Spain;* ^7^ *Adipocyte and Fat Biology Laboratory (AdipoFat), Translational Research Unit, University Hospital Miguel Servet, Instituto Aragonés de Ciencias de la Salud (IACS), Zaragoza, Spain;* ^8^ *Centro de Investigación Biomédica en Red Fisiopatología Obesidad y Nutricion (CIBERObn), Instituto Salud Carlos III, Madrid, Spain*

Anthropometry is an easily obtainable and non‐invasive method to evaluate the nutritional status in institutionalized elderly people, who are often bedridden. We aimed to investigate the relationship between neck circumference (NC) and nutritional status in elderly nursing home residents and to find cut‐off points for NC size to identify individuals at risk of malnutrition within this population.

**Methods:** We enrolled 352 elderly people living in five public nursing homes for this cross‐sectional study. The Mini Nutritional Assessment (MNA) was used to determine the nutritional status and different anthropometric measures were evaluated. Local Ethical Committee approved.

**Results:** The mean age of the population (59% females) was 83 years; 48.3% of women and 45.5% of men were at risk of malnutrition according to their MNA scores. All anthropometric measurements were highly intercorrelated in both men and women, indicating a high degree of collinearity. Bootstrapped linear regression was used assess the strength of the association between individuals' nutritional status and anthropometric parameters. Calf circumference and neck circumference presented the best predictive value with the highest sensitivity for the diagnosis of risk of malnutrition in both institutionalized elderly men and women. The best cut‐offs of NC to identify individuals at risk of malnutrition were 35.2 cm for females and 37.8 cm for males.

**Conclusions:** NC is associated with other classical anthropometric parameters and with malnutrition status in elderly people living in nursing homes.

**6-18**

**Nutritional support in patients with incurable cancer: a systematic review**

**Honor Blackwood**, Erna Haraldsdottir, Charlie Hall and Barry Laird

*Centre for Education and Research, St Columba's Hospice, Edinburgh, UK*

**Introduction:** Recent European Society for Clinical Nutrition and Metabolism (ESPEN) guidelines have advocated increased attention to dietetic services including nutritional counselling to patients with cancer; however, little is known about the optimal nutritional support, including specific interventions. Therefore, the aim of this systematic review was to assess the current evidence for nutritional support via dietary interventions in patients with incurable cancer.

**Methods:** A systematic review was conducted in accordance with the Preferred Reporting Items for Systematic Reviews (PRISMA) guidelines. Medline, PubMed, and CIHNAL were searched from 1990–2018. Eligible studies examined nutritional interventions in adult patients with incurable cancer.

**Results:** Sixty‐five studies were examined of which eight met the eligibility criteria. Of the eligible studies, six were randomized controlled trials and two were observational studies. Two studies examined weight as a primary endpoint, of which both reported statistically significant improvements in weight. Three studies examined quality of life as a primary endpoint, and all three reported that nutritional interventions improved quality of life. Only one study examined lean mass as an endpoint but demonstrated significant improvements. The most common nutritional interventions examined were nutritional counselling and dietary supplementation.

**Conclusions:** There is moderate evidence to support increased attention to nutritional support in patients with incurable cancer. Key questions remain as to the optimal time for these interventions to be implemented (e.g. cachexia stage, illness stage, and alongside anticancer therapy), appropriate endpoint measures and considerations of future study design.

**6-19**

**Cross‐sectional associations of the timing and variability of daily energy intake with parameters of body composition in long‐term colorectal cancer survivors**

**Martijn J.L. Bours**, Stevie Hendriks, Janna L. Koole, Eline H. van Roekel, José J.L. Breedveld‐Peters and Matty P. Weijenberg

*Department of Epidemiology, GROW – School for Oncology and Developmental Biology, Maastricht University, PO Box 616, Maastricht, The Netherlands*

**Background:** An unhealthy body composition (obesity, visceral adiposity, and sarcopenia) contributes to colorectal cancer (CRC) development. Consequently, many CRC survivors have an unhealthy body composition. This is likely related to their dietary habits. Specific aspects of the diet are timing and variability of daily energy intake. These are the focus of chrononutrition studies on relations between time‐related dietary aspects, circadian rhythms, and metabolism. We aimed to study associations of the timing and variability of daily energy intake with parameters of body composition in CRC survivors.

**Methods:** Cross‐sectional study in 155 Stage I–III CRC survivors, 2–10 years post‐diagnosis. Data from 7‐day food diaries on energy intake at breakfast, lunch, dinner, and snacking in‐between regular mealtimes were used. Timing of daily energy intake was calculated as intake at separate mealtimes or snacking as percentage of total daily intake, reflecting time‐of‐day distribution of energy intake. Variability of daily energy intake over repeated days was expressed by an irregularity score, with higher scores reflecting more day‐to‐day variation in total energy intake and intake at separate mealtimes or snacking. Parameters of body composition were BMI, waist circumference, body fat percentage, and handgrip strength. Multiple linear regression was performed, adjusted for relevant confounders including age, sex, physical activity, and total energy intake.

**Results:** More variability of daily energy intake at breakfast was significantly (*P* < 0.05) associated with higher BMI and waist circumference. More variability of daily energy intake at breakfast and dinner and of total daily intake were significantly associated with higher handgrip strength. A higher percentage of total daily energy intake during lunch was also significantly associated with higher handgrip strength. No significant associations were observed for other body composition parameters.

**Conclusions:** Day‐to‐day variation in energy intake was associated with body composition in long‐term CRC survivors. Further research on longitudinal associations and underlying metabolic mechanisms is warranted.

**6-20**

**Fat is beautiful, weight loss is dangerous? Ad‐libitum diets may be nutritonally safe, under strict control, in CKD patients**

Luigi Teta^1^, Irene Capizzi^2^, Federico Gennaro^2^, Frederica Nerve Vigotti^3^, Antioco Fois^4^, **Antoine Chatrenet** ^4^ and Giorgina Barbara Piccoli^1,4^

^1^ *Bioimis Accademia Alimentare, Bassano del Grappa, Vicenza, Italy;* ^2^ *Department of Clinical and Biological Sciences, University of Torino, Torino, Italy;* ^3^ *Nephrology, Chivasso Hospital, Turin, Italy;* ^4^ *Néphrologie, Centre Hospitalier du Mans, Le Mans, France*

**Introduction:** Obesity management is a challenge in chronic kidney disease (CKD): weight loss is often needed to access the transplant wait list, while weight loss is associated with excess mortality on dialysis.

Impact of weight loss on nutritional status is unclear for CKD patients. In this context, we report a prospective observational and long‐term experience of integration of a coach assisted, ad‐libitum, qualitative approach to weight loss in the choice ‘menu’ of overweight or obese CKD patients.

**Methods:** Diet phase: start of diet: March 2013–December 2014; short‐term follow‐up: December 2015. Long‐term follow‐up: December 2017. Enrolment in a weight loss ad‐libitum, qualitative program (Bioimis) was proposed to all adult overweight patients followed in an Italian Unit (Charlson Index <10). Patients could choose between the study diet and a ‘traditional’ approach. Anthropometric, quality of life (SF‐36), reasons of choice, nutritional, dietary satisfaction (MDRD questionnaire), and kidney function data were recorded at different intervals. Forty non‐nephropathic patients enrolled in the ad‐libitum diet served as controls.

**Results:** Forty‐five CKD patients were enrolled (whose 27 ad‐libitum). No difference was present at baseline as for age, eGFR, proteinuria, BMI, and comorbidity, but control age was lower and BMI higher.

At the end of short‐term follow‐up, all groups attained a significant weight loss (ad‐libitum, BMI: −3.2 kg/m^2^; other diets: BMI −1.8 kg/m^2^; healthy controls: BMI −4.8 kg/m^2^).

At the end of follow‐up (3 years), only two CKD patients in each group had significantly gained weight while a decrease of −3 and −2 kg/m^2^ of BMI was confirmed in the others.

During follow‐up, main nutritional parameters remained stable, and a trend towards improvement of quality of life was observed.

**Conclusions:** Weight loss may be nutritionally safe in CKD patients under strict clinical surveillance; ad‐libitum diets may safely integrate the dietary options.

**6-21**

**Different countries, different diets: moderate protein restriction in Italy and France: different patient choice different diets**

**Antioco Fois** ^1^, Antoine Chatrenet^1,2^, Françoise Lippi^4^, Ana‐Kadij Kaniassi^4^, Ludivine Froger^4^, Jérome Vigreux^4^, Zineb Filali Khattabi^1^, Emanuela Cataldo^5^ and Giorgina and Barbara Piccoli^1,3^

^1^ *Néphrologie, Centre Hospitalier du Mans, Le Mans, France;* ^2^ *Laboratoire Motricité, Interactions, Performance, Le Mans, France;* ^3^ *Dipartimento di Scienze Cliniche e Biologiche, Università di Torino, Torino, Italy;* ^4^ *Diététique et Nutrition, Centre Hospitalier du Mans, Le Mans, France;* ^5^ *Nephrology, Università « Aldo Moro », Bari, Italy*

**Introduction:** Dietary background is a crucial determinant not only of health but also of interventions aimed at improving care of specific disease, including chronic kidney disease (CKD). Mediterranean diets are usually considered as easier to adapt to a low‐moderate protein‐restricted approach. The aim of the study is to compare dietary choice in two different settings, Italy and France, in patients followed with a similar multiple choice philosophy.

**Methods:** Choice of diets and patient features observed in two units allocated to advanced CKD in Italy and France were analysed (outcomes: compliance assessed with Mitch formula; choice according to protein intake side effect).

**Results:** The french population is older and with higher comorbidity. Baseline intake was probably higher (1.0–1.4 in France; 0.8–1.2 in Italy). In both settings, protein restriction was feasible, but normalization was more often demanded in France (57%), and vegan‐vegetarian diets were more often chosen in Italy (59.6%), while traditional diets were preferred in France (18%). The availability of protein‐free food was a crucial tool allowing protein restriction in Italy, not available in France; supplementation with alfa‐keto analogues was employed in both settings in about 50% of moderately restricted diets. Compliance reached 75% in France and around 80% of patients in Italy with ≥3 months on diets. No sign of protein malnutrition was observed in stable patients (Table 1).

**Table 1:** Comparison between France and Italian population

First diet	French cohort (UIRAV)	Italian cohort	*P*
*n*	131	457	
Males, *n* (%)	82 (62.6%)	281 (68.5%)	0.818
Females, *n* (%)	49 (37.4%)	176 (38.5%)	
Age, median (min–max)	74 (24–101)	70 (19–97)	0.124
Age over 65, *n* (%)	95 (72.6%)	274 (60%)	**0.010**
Age over 80, *n* (%)	49 (37.4%)	73 (15.4%)	**0.000**
Charlson, median (min–max)	8 (2–14)	7 (2–13)	**0.018**
Charlson ≥7, *n* (%)	93 (71%)	248 (54.8%)	**0.001**
Charlson ≥10, *n* (%)	22 (16.8%)	71 (15.5%)	0.728
Diabetes, *n* (%)	66 (50.4%)	150 (32.8%)	**0.000**
Cardiopathy, *n* (%)	30 (22.9%)	210 (46%)	**0.000**
Neoplasia, *n* (%)	17 (13%)	99 (21.7%)	**0.028**
BMI (kg/m^2^), median (min–max)	28.3 (16.7–51.2)	26.1 (13.3–51.4)	0.115
Creatinine (mg/dL), median (min–max)	2.6 (1.3–10.4)	2.8 (0.5–74)	0.225
eGFR‐EPI (mL/min), median (min–max)	22 (4–68)	20 (3–127)	0.302
GFR <15 (mL/min) at enrolment, *n* (%)	24 (18.3%)	125 (27.4%)	**0.036**
GFR <10 (mL/min) at enrolment, n (%)	11 (8.4%)	44 (9.8%)	0.670
Proteinuria (g/day), median (min‐max)	0.5 (0.1–8.3)	0.80 (0.1–12)	0.09
Proteinuria ≥1 g/day, *n* (%)	50 (38.2%)	203 (44.4%)	0.203
Proteinuria ≥3 g/day, *n* (%)	22 (16.8%)	79 (17.3%)	0.895
Glomerulonephritis‐systemic disease, *n* (%)	3 (2.3%)	95 (21.2%)	**0.000**

**Conclusions:** Protein restriction is feasible in different settings, even in high comorbidity populations, provided that the system is adapted to local needs.

**6-22**

**A personalized protein‐restricted nutritional approach in an old and high comorbidity population**

**Antioco Fois** ^1^, Antoine Chatrenet^1,2^, Françoise Lippi^4^, Ana‐Kadij Kaniassi^4^, Ludivine Froger^4^, Jérome Vigreux^4^, Sylvain Durand^2^, Bruno Beaune^2^, Zineb Filali Khattabi^1^ and Giorgina and Barbara Piccoli^1,3^

^1^ *Néphrologie, Centre Hospitalier du Mans, Le Mans, France;* ^2^ *Laboratoire Motricité, Interactions, Performance, Le Mans, France;* ^3^ *Dipartimento di Scienze Cliniche e Biologiche, Università di Torino, Torino, Italy;* ^4^ *Diététique et Nutrition, Centre Hospitalier du Mans, Le Mans, France*

**Introduction:** Protein restriction is a valuable tool for stabilizing chronic kidney disease (CKD) and retarding the need for renal replacement therapy. Old age and comorbidity are perceived as barriers, considering the risk of malnutrition and muscle wasting. Baseline dietary habits, especially in elderly patients, are considered difficult to change.

**Methods:** In a newly organized unit for advanced CKD (UIRAV) in France, we personalized the nutritional approach in order to normalizing‐reducing protein intake, in patients without malnutrition, advanced neoplasia, and short life expectancy. Different options were offered to patients with CKD Stages 3–5, not on dialysis: normalization of protein intake (0.8 g/kg/day), supplemented by ketoacids in case of nephrotic proteinuria; moderate protein restriction (0.6 g/kg/day), with a ‘traditional’ mixed protein or ‘plant based’ diet, usually supplemented with ketoacids. Compliance was estimated by Maroni Mitch formula and dietary journal, and nutritional status by SGA and biochemical markers.

**Results:** One hundred thirty‐one patients with CKD Stages 3–5 were studied [baseline protein intake 1.2 g/kg/day; age median 74 (24–101); comorbidity: Charlson index: 8 (2–14); eGFR 22 (4–68) mL/min; 50.4% diabetics, 40.5% had a BMI over 30 kg/m^2^], protein restriction was not proposed to 10 patients. Normalization of protein intake was the target in 75 patients (median age 78, Charlson 8, eGFR 24). Moderate protein restriction was chosen in 46 cases (‘traditional’ in 24: age 74, Charlson 8, GFR 22; ‘plant based’ in 22: age 70, Charlson 6.5, GFR 15). Adherence, evaluated in 65 patients with follow‐up ≥3 months, was good in about 75% of the cases (Table 1). Nutritional parameters remained stable or improved (serum albumin increased by ≥0.1 g in all subsets), and no patient discontinued the diet because of malnutrition.

**Conclusions:** Moderate protein restriction is feasible and nutritionally safe, even in elderly, high comorbidity patient. A stepwise multiple‐choice approach allows good adherence.

**6-23**

**Chlorella intake prevents aging‐induced muscle atrophy and fibrosis in senescent mice**

**Naoki Horii** ^1,2^, Masataka Uchida^1^, Natsuki Hasegawa^1^, Shumpei Fujie^2,3^, Toru Mizoguchi^4^, Eri Okumura^4^, Kiyoshi Sanada^1^ and Motoyuki Iemitsu^1^

^1^ *Ritsumeikan University, Kyoto, Japan;* ^2^ *Japan Society for the Promotion of Science, Tokyo, Japan;* ^3^ *University of Tsukuba, Tsukuba, Japan;* ^4^ *Sun Chlorella Corp*

**Introduction:** Muscle mass and strength is decreased by aging. Aging‐induced loss of muscle mass is associated with the enhancements of muscle protein degradation and muscle fibrosis. Our recent study showed that resistance training increases muscle mass and strength concomitant with inhibition of muscle fibrosis in senescent mice. Although chlorella has a potential to lead to muscle hypertrophy in adult mice, it is still unclear whether chlorella intake prevents aging‐induced muscle atrophy and fibrosis. Therefore, the aim of this study was investigated whether chlorella intake prevents aging‐induced muscle atrophy and fibrosis in senescent mice.

**Methods:** Male 13‐week‐old SAMP1 mice (Young) and 38‐week‐old SAMP1 mice (Aged) were randomly divided into four groups: young‐sedentary control (Young‐Con), aged‐sedentary control (Aged‐Con), aged‐chlorella intake (Aged‐CH; 0.5% chlorella powder in normal feed), and aged‐resistance training (Aged‐RT; climbing ladder 3 days/week) groups for 12 weeks (*N* = 8 per group). Cross‐sectional area (CSA) and fibrosis area in tibialis anterior (TA) muscle were measured by masson trichrome stain. Muscle strength was defined as the maximal amount of tail weight, which could perform a single climbing.

**Results:** Muscle strength, TA muscle mass, CSA in TA muscle, muscle strength per CSA in TA muscle significantly decreased in the Aged‐Con group as compared with the Young‐Con group (*P* < 0.05), whereas those in the Aged‐CH and Aged‐RT groups significantly increased as compared with the Aged‐Con group (*P* < 0.05). Muscle fibrosis area significantly increased in the Aged‐Con group as compared with the Young‐Con group (*P* < 0.05), whereas that in the Aged‐CH and Aged‐RT groups significantly decreased as compared with the Aged‐Con group (*P* < 0.05). However, these improvement effects in the Aged‐CH group were significantly smaller than those in the Aged‐RT group (*P* < 0.05).

**Conclusions:** These results suggest that chlorella intake prevents aging‐induced muscle atrophy and fibrosis in senescent mice, but less effective than resistance training.

**6-24**

**Acute effects of oral triglyceride load on endothelial function in patients with chronic heart failure**

**Azadeh Shafieesabet** ^1^, Nadja Scherbakov^1,2^, Stefanie Lokau^3^, Nicole Ebner^4^, Stephan von Haehling^4^, Stefan D. Anker^5,6^ and Wolfram Doehner^1,2,5,6^

^1^ *Center for Stroke Research Berlin, Charite Universitätsmedizin Berlin, Berlin, Germany;* ^2^ *German Centre for Cardiovascular Research (DZHK), Partner Site Berlin, Germany;* ^3^ *Applied Cachexia Research, Charité – Universitätsmedizin Berlin, Germany;* ^4^ *Department of Cardiology and Pneumology, University of Göttingen, Göttingen, Germany;* ^5^ *Department of Cardiology, Charite University Medical School, Berlin, Germany;* ^6^ *Berlin‐Brandenburg Center for Regenerative Therapies (BCRT), Charité ‐ Universitätsmedizin Berlin, Berlin, Germany*

**Background:** Postprandial hyperlipemia impairs endothelial function possibly via oxidative stress mechanisms. The aim of this study was to evaluate acute effects of an oral triglyceride load on endothelial function in patients with chronic heart failure (CHF) compared to healthy controls.

**Methods:** We enrolled 47 patients with CHF (age 64.3 ± 9.9 years, BMI 27.9 ± 5.4 kg/m^2^, NYHA 3.2 ± 1.4) and 20 healthy controls of similar age and BMI. Patients were in stable compensated condition on standard individually adjusted medical therapy with the history of CHF at least 6 months.

Peripheral endothelial function was assessed by EndoPAT2000 technology using reactive hyperemia index (RHI) and pulse wave amplitude (PWA) at baseline (after 8 h overnight fasting) as well as 1, 2, 3, and 4 h after oral triglyceride load (200 mL of cream 20%). Post cream index (PCI) was calculated as a ratio of PWA at each time point to baseline PWA.

**Results:** RHI at baseline was lower in CHF patients compared to controls (1.7 ± 0.7 vs. 2.3 ± 0.6, respectively, *P* < 0.001). After 4 h, PCI significantly increased in healthy controls but not in patients with CHF (1.9 ± 1.3 vs. 1.0 ± 0.5, respectively, *P* < 0.05). Triglyceride serum level increased from baseline to 4 h in both, patients with CHF (1.6 ± 1.0 to 3.0 ± 1.8 mmol/L, *P* < 0.001) and in controls (1.11 ± 0.5 to 2.2 ± 1.2 mmol/L, *P* < 0.001).

**Conclusions:** The postprandial vasodilation as observed in healthy controls was impaired in patients with CHF. In fasting condition and after oral lipid load, patients with CHF have impaired endothelial function. Normal endothelial function is markedly impaired by a high‐fat load that causes an acute hypertriglyceridemia. This impairment is evident in patients with CHF but not in healthy subjects.
